# A Link between ORC-Origin Binding Mechanisms and Origin Activation Time Revealed in Budding Yeast

**DOI:** 10.1371/journal.pgen.1003798

**Published:** 2013-09-12

**Authors:** Timothy Hoggard, Erika Shor, Carolin A. Müller, Conrad A. Nieduszynski, Catherine A. Fox

**Affiliations:** 1 Department of Biomolecular Chemistry, School of Medicine and Public Health, University of Wisconsin-Madison, Madison, Wisconsin, United States of America; 2 Program in Cellular and Molecular Biology, College of Agriculture and Life Sciences, University of Wisconsin-Madison, Madison, Wisconsin, United States of America; 3 Centre for Genetics and Genomics, University of Nottingham Queen's Medical Centre, Nottingham, United Kingdom; University of California San Francisco, United States of America

## Abstract

Eukaryotic DNA replication origins are selected in G1-phase when the origin recognition complex (ORC) binds chromosomal positions and triggers molecular events culminating in the initiation of DNA replication (a.k.a. origin firing) during S-phase. Each chromosome uses multiple origins for its duplication, and each origin fires at a characteristic time during S-phase, creating a cell-type specific genome replication pattern relevant to differentiation and genome stability. It is unclear whether ORC-origin interactions are relevant to origin activation time. We applied a novel genome-wide strategy to classify origins in the model eukaryote *Saccharomyces cerevisiae* based on the types of molecular interactions used for ORC-origin binding. Specifically, origins were classified as DNA-dependent when the strength of ORC-origin binding in vivo could be explained by the affinity of ORC for origin DNA in vitro, and, conversely, as ‘chromatin-dependent’ when the ORC-DNA interaction in vitro was insufficient to explain the strength of ORC-origin binding in vivo. These two origin classes differed in terms of nucleosome architecture and dependence on origin-flanking sequences in plasmid replication assays, consistent with local features of chromatin promoting ORC binding at ‘chromatin-dependent’ origins. Finally, the ‘chromatin-dependent’ class was enriched for origins that fire early in S-phase, while the DNA-dependent class was enriched for later firing origins. Conversely, the latest firing origins showed a positive association with the ORC-origin DNA paradigm for normal levels of ORC binding, whereas the earliest firing origins did not. These data reveal a novel association between ORC-origin binding mechanisms and the regulation of origin activation time.

## Author Summary

Cell division requires the duplication of chromosomes, protein-DNA complexes harboring genetic information. Specific chromosomal positions, origins, initiate this duplication. Multiple origins are required for accurate, efficient duplication—an insufficient number leads to mistakes in the genetic material and pathologies such as cancer. Origins are chosen when the origin recognition complex (ORC) binds to them. The molecular interactions controlling this binding remain unclear. Understanding these interactions will lead to new ways to control cell division, which could aid in treatments of disease. Experiments were performed in the eukaryotic microbe budding yeast to define the types of molecular interactions ORC uses to bind origins. Yeasts are useful for these studies because chromosome duplication and structure are well conserved from yeast to humans. While ORC-DNA interactions were important, interactions between ORC and chromosomal proteins played a role. In addition, different origins relied on different types of molecular interactions with ORC. Finally, ORC-protein interactions but not ORC-DNA interactions were associated with enhanced origin function during chromosome-duplication, revealing an unanticipated link between the types of molecular interactions ORC uses to select an origin and the ultimate function of that origin. These results have implications for interfering with ORC-origin interactions to control cell division.

## Introduction

Eukaryotic DNA replication initiates at specific chromosomal sites called origins. An origin is selected in G1-phase by the origin recognition complex (ORC) that directly binds chromosomal DNA, triggering a series of molecular events that culminate in the loading of an MCM helicase complex onto DNA (reviewed in [1]–[4]). Origin activation (unwinding; firing) occurs only during the subsequent S-phase, when the MCM complex is activated to give two oppositely oriented helicases that will unwind DNA at the bidirectional replication forks (reviewed in [5]–[7]). Temporal separation of the origin selection and activation steps helps ensure a chromosome is replicated only once per cell cycle (reviewed in [8]). However, it is unclear how the specific molecular events essential for the first step, in particular origin binding by ORC in G1 phase, might regulate the second step, origin activation in S-phase.

While the understanding of the roles of origin-binding factors has progressed to a mechanistic level, we have less understanding of how the firing of eukaryotic replication origins is regulated during S-phase. In particular, a eukaryotic chromosome requires the action of multiple origins for its timely and accurate replication [9]–[13]. Individual origins vary in their time of activation during S-phase, creating a distinct spatial and temporal pattern of genome duplication that, in multicellular organisms, shows cell-type specificity and is associated with normal cell differentiation. Indeed, disruption of replication timing contributes to genome instability [14]–[17]. The conservation of replication timing patterns—for example histone genes and centromeres are replicated early during S-phase, while telomeres are replicated late in many organisms—and their strong association with genome stability and differentiation have spurred research to define the mechanisms that control origin activation time [18]–[21]. Despite important advances, including several reported in recent studies, the specific molecular features of DNA replication origins that control their activation time remain incompletely understood [22]–[27].

Many studies examining origin activation time have used the model eukaryote budding yeast *S. cerevisiae* and dealt with factors with broad effects on DNA replication and other chromosomal processes, such as S-phase kinases, Forkhead family transcription factors, and chromatin structure [22], [28]–[30]. Indeed, early studies in yeast established that chromosomal context could have a substantial impact on an origin's activation time [31], [32]. For example, an origin that normally fires early could be made to fire late by placing it within a region of heterochromatin, while specific modifications associated with actively transcribed chromatin, such as histone acetylation, could advance an origin's replication time in both yeast and flies [33]–[36]. Thus chromatin structure can clearly regulate origin activation time, although in many cases the molecular step affected is unknown.

Although a major focus of the timing studies has been on factors extrinsic to core origin function, a few studies have raised the possibility that origin-binding factors, required for origin activation *per se*, can influence origin activation time. In particular, recent studies reveal that a collection of origin-activation factors are limiting in S-phase such that their over-expression can advance the replication time of a normally late-firing origin [23], [24]. This observation raises questions about the mechanisms that underlie the differential affinities of origins for these limiting factors. Although specific chromatin structures likely contribute, it is unclear how they affect core factors that establish the origin-protein complex that is recognized during S-phase—such as ORC or the MCM complex [37].

In *S. cerevisiae*, ORC selects origins in part by interacting in a sequence-specific manner with a conserved DNA element present in all yeast origins [38], [39]. At the simplest level, one might predict that stronger interactions between ORC and its binding site would enhance origin activity and therefore contribute to earlier, more robust origin activation during S-phase. Indeed, a study of *S. pombe* origin activation time provides evidence in support of this idea [40]. However, a previous study from our group revealed that the relationship between ORC-origin interactions and origin activation time might be more complex [41]. In particular the ORC binding site within a specific origin, *ARS317*, also known as the *HMR*-E silencer that controls heterochromatin formation at the silent mating-type locus *HMR*, binds ORC with a remarkably high affinity in vitro compared to several other replication origins. A noteworthy characteristic of *HMR*-E, in addition to its silencer function, is that it functions extremely poorly as a replication origin, firing in only a small percentage of cell cycles and then only very late in S-phase (reviewed in [42]). Thus, paradoxically, the high-affinity ORC-origin interaction at *ARS317* fails to promote efficient or early origin activation. In fact, mutations that weaken the ORC-DNA interaction enhance the firing efficiency and advance the replication time of *ARS317*. In addition, several efficient and early activating origins examined in the same study have weak ORC-DNA interactions in vitro. Thus a high-affinity ORC-origin interaction mediated by sequence-specific ORC-DNA contacts is insufficient to promote - and can in fact inhibit - robust, early origin activation. However, we examined only a small number of yeast origins in this study, leaving in question whether this conclusion could be extended to form a general paradigm about the relationship between ORC-origin interactions and origin firing.

An additional complication is revealed by recent studies establishing that, in addition to sequence-specific interactions between ORC and origin DNA, chromatin also contributes to yeast ORC's ability to bind origins, as it does for metazoan ORC [43]–[45]. For example, the bromo adjacent homology (BAH) domain of Orc1, a nucleosome-binding domain within the largest subunit of ORC, contributes to ORC-origin binding in both yeast and mammalian cells [43], [46], [47]. However, individual yeast origins vary substantially in terms of their requirement for the Orc1BAH domain for ORC binding, suggesting that the mechanisms governing ORC-origin binding in budding yeast vary between origins, with some, such as *ARS317* using sequence-specific ORC-DNA interactions and others using as yet incompletely defined ORC-chromatin interactions [43]. Thus the stability of an ORC-origin complex in vivo could be achieved by sequence-specific ORC-origin DNA interactions, as at *ARS317*, or by interactions between ORC and accessory proteins (e.g. chromatin), or by some combination of these mechanisms. It is entirely unknown whether these different mechanisms of ORC-origin binding might ultimately relate to the regulation of origin activation in S-phase.

In this study we addressed these issues by employing a genome-wide approach to determine the relative contribution to ORC-origin binding of ‘intrinsic’ features (i.e. the DNA sequence that comprises the ORC binding site) versus ‘extrinsic’ features (e.g. adjacent ‘chromatin’, including both nucleosomes and non-histone proteins). This approach allowed us to classify origins into several groups based on the type of mechanism that stabilized the ORC-origin complex. We focused further comparative analysis on two distinct groups of origins, each of which bound ORC with similar strengths in vivo. The first group was comprised of DNA-dependent origins, such as *ARS317*, in which the DNA sequence of the ORC binding site was a primary determinant of ORC-origin affinity. The second group was comprised of ‘chromatin-dependent’ origins in which the ORC-origin DNA interaction was insufficient to explain the ORC binding strength in vivo. As expected from a biologically meaningful classification, DNA-dependent and ‘chromatin-dependent’ origins differed based on several other structural and functional criteria. Significantly, the ‘chromatin-dependent’ group was enriched with origins that fire early in S-phase whereas the DNA-dependent group that included *ARS317* was enriched for later firing origins. Moreover, the latest-firing origins in the genome, as a group, showed a positive correlation between in vivo and in vitro ORC-origin binding affinity, indicating that many of these origins followed the DNA sequence-dependent ORC-origin interaction paradigm. In contrast, the earliest-firing origins, as a group, showed no correlation between in vivo and in vitro ORC-origin binding affinity, suggesting that ‘chromatin’ had a larger impact on ORC-origin binding at many of the earliest firing origins. Taken all together these data provided evidence that sequence-specific ORC-DNA interactions that promote ORC-origin binding stability are often associated with the suppression of origin activation, whereas ORC-‘chromatin’ interactions that modulate this stability are often associated with the enhancement of origin activation. We discuss the interesting mechanistic implications of this unanticipated connection between the mode of ORC-origin binding and origin activation time.

## Results

### Classifying yeast origins based on the contribution of the ORC-origin-DNA interface to the strength of the ORC-origin interaction in vivo

**Figure 1:**
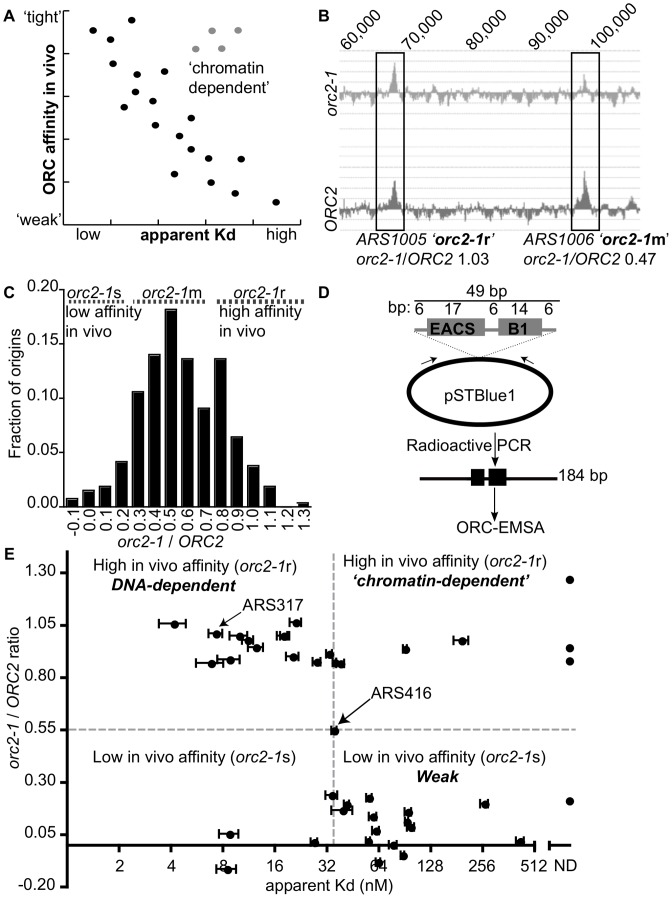
Classifying yeast origins based on the contribution of the ORC-origin-DNA interface to the strength of the ORC-origin interaction in vivo. (A) If the strength of ORC-origin interactions in vivo were due to interactions between ORC and the essential ORC binding site within origins, then we would expect a graph in which in vivo ORC-origin binding strengths (x-axis) plotted against in vitro ORC-origin-DNA binding strengths (apparent Kd) to show a correlation (black dots). Such a correlation would allow us to identify ‘exceptions to the rule’ such as those shown as putative ‘chromatin-dependent’ origins (gray dots). (B) ORC ChIP peaks are shown for two adjacent origins on chromosome X, *ARS1005*, and A*RS1006*. The relevant chromosomal coordinates are shown at the top of the figure. *ARS1005* has a high-affinity for ORC in vivo (*orc2-1*-resistant *(orc2-1*r)) relative to *ARS1006* that has a moderate affinity for ORC in vivo (*orc2-1*-moderately sensitive (*orc2-1*m)). (C) The fraction of the confirmed origins defined in our wild-type (*ORC2* ChIP-chip) array (n = 261; y-axis) was plotted against the corresponding *orc2-1/ORC2* ratios (x-axis). For a small number of origins the corresponding genomic region in *orc2-1* cells showed a slight depletion in ORC binding and hence generated a negative value of −0.1. (D) Schematic of the DNA probes used in EMSAs with ORC. All probes contained the confirmed or predicted ORC binding site in the orientation shown for each origin listed in Table 1. (E) Scatter plot comparing the ORC-origin binding affinities in vivo (*orc2-1/ORC2* (y-axis)) and in vitro (apparent Kd (x-axis)). The average apparent Kd and standard error obtained from three independent experiments are shown. *ARS317* (*HMR*-E) and *ARS416* (*ARS1*) known to bind ORC with high- and moderate- affinity in vitro, respectively, are indicated [41].

The paradigm for yeast origin selection by ORC is that sequence-specific ORC-origin-DNA (hence referred to as ORC-DNA) interactions drive this process. Specifically, ORC binds to a bipartite ∼35 bp element consisting of a 17-bp EACS-element (extended ARS consensus sequence) and a less conserved B1-element that contains a common 3 bp WTW motif [48]–[50]. However, recent work has shown that local chromatin structure may also contribute to origin selection by ORC [43], [44]. This observation raises the possibility that some yeast origins might rely primarily on ORC-‘chromatin’ interactions for ORC binding while other origins rely on sequence-specific ORC-DNA interactions. If the sequence-specific ORC-DNA interaction controls ORC binding at many yeast origins in vivo, then we would expect a correlation between in vivo and in vitro ORC-origin binding strengths, whereas origins that deviated from this correlation would be putative ‘chromatin-dependent’ origins in which features extrinsic to the ORC binding sequence likely modulate origin-binding by ORC in vivo (Figure 1A). (The term ‘chromatin’ is used here in its broadest sense to include both histone- and non-histone-chromosomal proteins. Therefore at ‘chromatin-dependent’ origins we envision that ORC binds the origin locus through direct contacts with histones or non-histone chromosomal proteins such as transcription factors). Therefore we examined ORC-origin affinity in vivo and in vitro for a large fraction of yeast origins to classify them based on which mechanism (i.e. DNA sequence vs. extrinsic, ‘chromatin’ factors) mediates their binding to ORC in vivo.

To measure ORC's affinity for confirmed origins in vivo, we used data from an experiment in which ORC occupancy, as measured by ChIP, was compared genome-wide between wild-type and *orc2-1* mutant cells [51]. *Orc2-1* is a temperature-sensitive allele that reduces the amount of Orc2, an essential subunit of ORC, by ∼10-fold even at permissive growth temperatures. *Orc2-1* cells grow more slowly than wild-type cells and exhibit additional defects even at permissive temperatures [52]. However, simply over-producing *orc2-1* is sufficient to rescue these growth defects, including temperature-sensitivity, suggesting that the primary defect is reduction in levels of ORC. Moreover, detailed analyses of *ARS317*, the *HMR*-E silencer origin, reveals that mutations in the ORC binding site of this origin that enhance ORC-origin binding affinity in vitro, fully suppress the defects in this origin caused by the *orc2-1* allele. Collectively these data provide evidence that the primary defect caused by the *orc2-1* mutation is reduced ORC, such that ORC concentration becomes limiting in the nucleus [41], [51]. Thus, as expected, in the *orc2-1* mutant, an origin with high in vivo affinity for ORC remains fully occupied by ORC as measured by ChIP, while an origin with a low in vivo affinity shows reduced occupancy (Figure 1B and [51]). Therefore, we used the ratio of the areas of origin-associated ORC binding peaks in *orc2-1* mutant cells to that in wild-type cells (hence referred to as *orc2-1/ORC2* ratio) as a measure of ORC's ‘affinity’ for that origin in vivo. The *orc2-1/ORC2* ratio for every peak identified in the wild-type array that was associated with a confirmed origin in the yeast origin database (261/351 confirmed origins; [53]; http://cerevisiae.oridb.org) is plotted in Figure 1C. The confirmed origins exhibit a range of *orc2-1/ORC2* ratios. To aid in further analyses, we arbitrarily divided the origins into three groups: origins with *orc2-1/ORC2* ratios</ = 0.3 were termed ‘low in vivo affinity’ and considered *orc2-1*-sensitive (*orc2-1*s; n = 35); origins with *orc2-1/ORC2* ratios greater than 0.3 but less than 0.8 were termed ‘moderate in vivo affinity’ and considered *orc2-1*-moderately sensitive (*orc2-1*m; n = 175); origins with *orc2-1/ORC2* ratios of >/ = 0.8 were termed ‘high in vivo affinity’ and considered *orc2-1*-resistant (*orc2-1*r; n = 51).

Our goal was to distinguish between origins that used ORC-DNA interactions to achieve normal ORC binding in vivo from origins that used ORC-‘chromatin’ interactions. An expectation was that the established ORC-DNA interaction explained the ORC binding strength for many origins in vivo, as it did for *ARS317* and *ARS1 (ARS416)*. Thus if the in vivo-in vitro affinity correlation ‘rule’ for budding yeast origins was followed for some origins, we could be more confident that ‘exceptions to the rule’ would provide useful insights. As a proof-of-principle experiment, we selected 18 origins from among the lowest *(orc2-1/ORC2* ratios</ = 0.3) and 20 origins among the highest (*orc2-1/ORC2* ratios>/ = 0.8) in vivo affinity groups and determined the strength of the interaction between purified origin-DNA and purified ORC in vitro (Table 1). *ARS416 (ARS1)* was used to represent the ‘moderate in vivo affinity’ group. The ORC binding site for each of these 39 origins was cloned into a bacterial plasmid, and plasmid-specific PCR primers were used to generate radiolabeled DNA fragments of 184 bp with the ORC binding site centered within the fragment and arranged in the orientation shown (Figure 1D). Data from standard Electrophoretic Mobility Shift Assays (EMSAs) were used to measure ORC binding to these elements and calculate apparent Kds for each ORC-DNA complex. *ARS317* whose affinity for ORC in vivo could be accounted for by the strength of its ORC-DNA interaction was used as an internal standard in every EMSA [41], [51].

**Table 1:** ORC binding data for the 39 origins used in ORC binding reactions in Figure 1E.

origin	*orc2-1/ORC2*	apparent Kd(nM)	ACS status
728	−0.112	8.38+/−1.11	Confirmed^1^
1325	−0.079	63.04+/−1.943	Confirmed
911	−0.046	87.33+/−2.205	Confirmed
1329	0.004	76.8+/−4.002	Confirmed
1631	0.018	26.79+/−1.192	Confirmed
512	0.022	55.06+/−1.556	Confirmed
1413	0.022	418.9+/−21.76	Confirmed
920	0.057	8.652+/−1.116	Confirmed
604	0.072	61.03+/−2.888	Confirmed^2^
1405	0.09	97.18+/−4.689	Confirmed
1004	0.114	92.27+/−2.121	Confirmed^3^
1323	0.139	58.62+/−3.024	Confirmed
818	0.161	93.96+/−4.237	Confirmed
716	0.169	39.21+/−5.439	Confirmed
214	0.198	41.33+/−1.409	Confirmed
822	0.201	260.3+/−13.74	Confirmed
809	0.216	ND	Confirmed
1215	0.229	55.8+/−2.055	Predicted^a^
720	0.242	33.9+/−2.715	Confirmed
416	0.551	35.07+/−1.223	Confirmed^4^
824	0.871	38.11+/−2.034	Predicted^b^
423	0.872	6.757+/−1.214	Confirmed
1332	0.876	35.29+/−0.8786	Confirmed
516	0.878	27.76+/−1.439	Confirmed
105	0.885	ND	Confirmed
1625	0.889	8.669+/−1.295	Confirmed
201	0.903	20.13+/−1.65	Predicted^c^
1601	0.916	32.75+/−1.287	Predicted^c^
1521	0.94	90.13+/−2.361	Confirmed
1528	0.947	ND	Confirmed^5^
1123	0.948	12.35+/−1.231	Confirmed
1529.5	0.98	191.6+/−18.98	Confirmed^5^
1011	0.982	11.07+/−0.8141	Confirmed^3^
1021	1.003	17.9+/−0.8416	Confirmed^1^
1420	1.003	17.82+/−1.48	Confirmed
422	1.003	9.853+/−1.168	Confirmed
317	1.014	7.223+/−0.6519	Confirmed^6^
514	1.06	4.116+/−0.7319	Confirmed
1320	1.067	20.95+/−1.617	Confirmed
219.5	1.273	ND	Confirmed

The ORC binding site is the only element within origins essential for ARS function. Therefore if an ORC binding site is ‘confirmed’ it means that it has been shown experimentally that a mutation in this site (specifically the ACS, which is the most conserved part of the A-element) abolishes ARS function. The majority of ORC binding sites listed in this table were either confirmed previously (primary reference listed) or confirmed for this study. Notes and Citations:

a : Predicted nimACS and proACS are equivalent;

b : Subtelomeric origin, ACS not confirmable in assay;

c : Telomeric ARS, ACS not confirmable in assay.

1 : [38];

2 : [91];

3 : [92];

4 : [93];

5 : [94];

6 : [48].

ND means that binding was too weak to determine an apparent Kd.

The *orc2-1/ORC*2 ratio of each examined origin was plotted against the apparent Kd determined from the EMSA experiments (Figure 1E). Many of these origins followed the yeast ORC-DNA paradigm in that their affinities for ORC in vivo correlated with their ORC-DNA affinities measured in vitro. For example, 11 of the 20 ‘high in vivo affinity’ origins bound ORC with relatively low Kds in vitro, indicating a high-affinity ORC-DNA interaction. We will refer to origins within this class as DNA-dependent because their high affinity for ORC in vivo correlated with a strong ORC-DNA interaction in vitro. Conversely, 14 of the 18 ‘low in vivo affinity’ origins bound ORC with relatively high Kds (apparent Kd>4x the Kd for the ORC-*ARS317* complex), indicating a low affinity ORC-DNA interaction. We will refer to origins within this class as Weak because their low affinity for ORC in vivo correlated with their weak ORC-DNA interaction in vitro. *ARS1*, the representative ‘moderate in vivo affinity’ origin bound ORC with a moderate Kd, as expected from previous work [41], [51].

As described above, for many origins tested, the ORC-DNA interaction was a good predictor of an origin's ‘in vivo affinity’ for ORC. However, there were several exceptions. For example, five ‘high in vivo affinity’ origins showed unexpectedly weak ORC-DNA interactions in vitro. In fact, for three of these (*ARS105*, *ARS219.5* and *ARS1528*) the confirmed ORC binding site bound ORC so poorly in vitro that an apparent Kd could not be determined. We will refer to origins that behave in this way as ‘chromatin-dependent’ because their high affinity for ORC in vivo did not correlate with their weak ORC-origin DNA interaction measured in vitro suggesting that features extrinsic to the ORC binding site—i.e. ‘chromatin’—were required for normal levels of ORC binding to these origins. The origin classifications defined above—DNA-dependent, ‘chromatin-dependent’ and Weak—will be used throughout this manuscript. Three of the 18 ‘low in vivo affinity’ origins also deviated from the paradigm's prediction, binding ORC more tightly in vitro than predicted, suggesting ‘chromatin’ played a negative role in ORC binding. While origins of this type were not pursued further in this study, they might be a consequence of transcription as reported previously [54], [55]. We note also that for both the ‘high and low in vivo affinity’ origin groups, there is a continuum in apparent Kd values. For example, a few origins in both groups bound ORC with moderate Kds in vitro, similar to that of *ARS1*, which has a ‘moderate in vivo affinity.’ We referred to these origins as complex to indicate that they use some combination of intrinsic ORC-DNA interactions and extrinsic ORC-‘chromatin’ interactions for normal levels of ORC binding in vivo.

### Extending comparisons of in vivo and in vitro ORC-origin interaction strengths genome-wide

**Figure 2:**
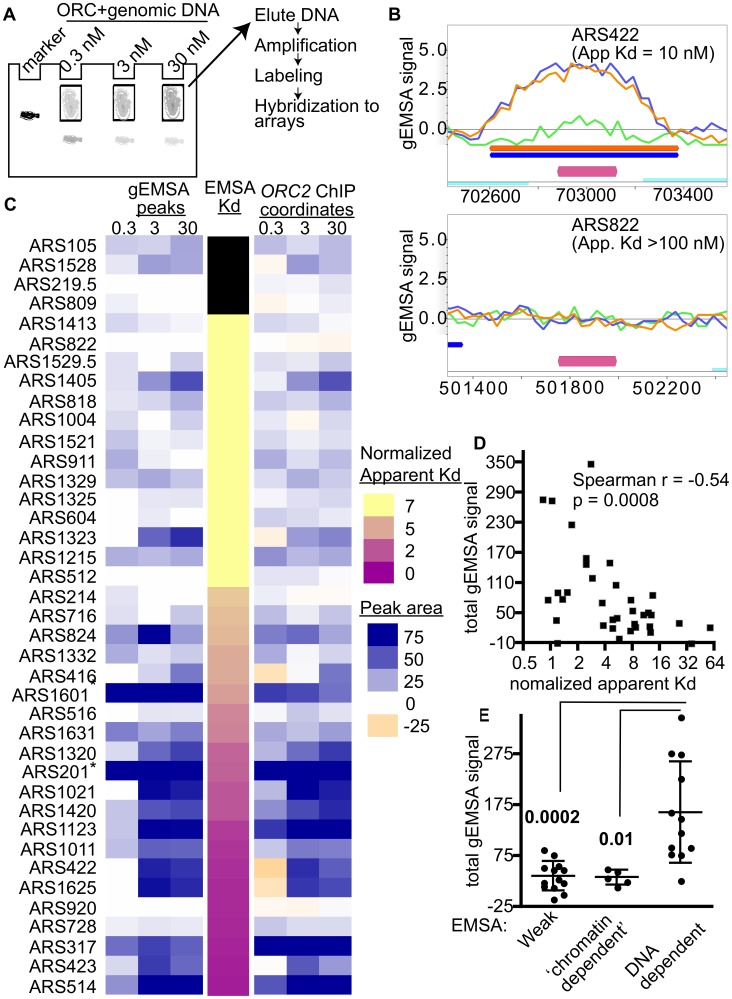
Extending comparisons of in vivo and in vitro ORC-origin interaction strengths genome-wide. (A) Outline of genomic EMSA: Purified ORC at the indicated concentrations was incubated with 0.5 pM of purified sheared yeast genomic DNA. After the reactions reached equilibrium, ORC-bound and -unbound DNA fragments were separated by native gel electrophoresis. To mark the position of ORC-DNA complexes in the gel, a control reaction was performed with a specific radiolabeled origin-containing fragment (184 bp marker). (B) ORC binding in the gEMSA was visualized in MochiView [88]. Screen shots are shown for two origins that were also examined by origin-specific EMSAs in Figure 1. The signals for each feature (oligo) on the array are indicated on the y-axis and the chromosomal coordinates are indicated on the x-axis. A magenta bar marks the coordinates comprising the oriDB annotated ARS. The signal strength for the 0.3 nM (green), 3 nM (blue) and 30 nM (orange) ORC concentrations are plotted and the coordinates corresponding to the ChIPotle called peaks at each concentration are shown by the correspondingly colored bars. The ORC-*ARS422* complex had an apparent Kd of 10 nM by origin-specific EMSAs (Figure 1E and Table 1), while the ORC-*ARS822* complex had an apparent Kd estimated to be >100 nM. (C) Heat maps depicting the ORC-origin binding strengths in the gEMSA for the 39 origins examined in Figure 1E or their normalized Kds (narrow map, center) are shown. gEMSA ORC-origin binding strength was defined two different ways, depending on the heat map. The heat map to the left (gEMSA peaks) defined the ORC-origin complex as the area of the peak that was called by the ChIPOTle program analysis of the gEMSA data in each independent array (ORC concentration shown at top of columns). If no binding peak was called, the ORC-origin binding strength was assigned a “0” value and colored white. The heat map on the right (*ORC2* ChIP coordinates) defined the ORC-origin complex in the gEMSA as the coordinates that comprised the ChIPOTle called ORC-origin peak for the previously published *ORC2* ChIP array [51]. Thus in this map, DNA regions that were depleted in the experimental sample relative to total DNA were assigned a negative value and colored beige. The two different approaches produced similar conclusions, suggesting that for most of these origins the gEMSA peak corresponded well to the in vivo ORC-origin peak. (D) Correlation analysis of gEMSA-derived binding strength versus normalized apparent Kds as determined by origin-specific EMSAs in Figure 1E. The normalized apparent Kd values were calculated by dividing apparent Kds by the apparent Kd for *ARS317* (*HMR*-E). The gEMSA-derived binding strength was a quantitative expression of the data shown in the right map in © as described above. Specifically, the gEMSA signal for each ORC concentration for each origin was summed to give a “Total gEMSA signal.” Thus this value simply expressed a value for total ORC-origin complex formation observed across the ORC titration. Our analysis included 35 origins for which apparent Kd values could be determined. A Spearman correlation coefficient and P-value are indicated. (E) We grouped origins into classes based on their EMSA derived apparent Kd values and their sensitivity to the *orc2-1* mutation for ORC binding in vivo. We compared the Total gEMSA signal for each of these three groups: weak *orc2-1*s (apparent Kd>5x *ARS317*, n = 13), weak *orc2-1*r (apparent Kd>10x *ARS317*, n = 5), and tight *orc2-1*r (apparent Kd</ = 3x *ARS317*, n = 12). T tests were used to determine the significance of the difference between the weak or ‘chromatin-dependent’ groups and the DNA-dependent group. P-values are indicated in figure.

To extend this approach to yeast origins on a genome-wide scale, we performed an EMSA using purified ORC (at 0.3, 3.0 and 30 nM) and purified, sheared yeast genomic DNA. We then screened the population of ORC-bound fragments by hybridizing the amplified and labeled DNA pool to tiled arrays (genomic EMSA or gEMSA) (Figure 2A). For each sample, ORC bound fragments were identified as binding peaks with a P-value selected to maximize the number of confirmed origins identified and to capture weaker ORC-DNA interactions (P-value>/ = 10^−5^). As a first step to determining the effectiveness of this approach, we examined the behavior of those origins we had analyzed with EMSAs (Figure 1E). Specifically, we examined the ORC binding signals generated by the gEMSA at each ORC concentration for each of these origins. Peaks associated with origins *ARS422* and *ARS822* are shown in Figure 2B, demonstrating how the gEMSA data recapitulated the origin-specific EMSA data for these ARSs.

Two different heat maps were generated to represent the strength of ORC-origin binding produced in the gEMSA for the 39 origins examined by EMSAs (Figure 2C). In one heat map the ORC-origin binding strength was defined as the area of the gEMSA peak called by ChIPOTle that overlapped the annotated ARS (Figure 2C, left panel labeled ‘gEMSA peaks’). The peak area was the sum of the signals for each feature (oligo) on the array that was included in the ChIPOTle-called peak. In the second heat map, ORC-origin binding strength was the sum of the signals of each oligo in the array that corresponded to the coordinates of the in vivo ORC binding peak from the ORC ChIP-chip experiment (Figure 2C, right panel labeled ‘*ORC2* ChIP coordinates’) [51]. The two approaches to defining ORC-origin binding strength in vitro produced similar results. Finally, these two representations of the gEMSA data were compared to origin-specific EMSA derived Kds (normalized to the Kd for the ORC-*ARS317* interaction; third narrow centrally positioned heat map). The 39 origins were ranked from weakest to strongest for ORC binding based on their normalized apparent Kds.

These analyses revealed that the gEMSA data recapitulated the origin-specific EMSA data well though not perfectly: in general the weakest ORC binding sites tested in vitro by EMSAs were associated with weaker binding signals in the gEMSA and vice versa (Figure 2C). Correlation analysis of the apparent Kds determined by EMSAs and the Total gEMSA signal (i.e. the sum of gEMSA ORC-origin binding strength for each ORC concentration) revealed significant co-variation (Spearman r coefficient = −0.54 and a P-value = 0.0008; Figure 2D). However, eight of the 39 origins examined did not produce gEMSA data that matched the predictions based on the EMSA-derived Kds. Two of these were telomeric ARSs that consistently produced broad peaks at all ORC concentrations tested. We removed core-X telomeric ARSs from further consideration in all subsequent bioinformatics analyses for this and other reasons. Three of these origins produced tighter binding in the gEMSA than predicted by the origin-specific EMSAs (*ARS1405*, *ARS1323* and A*RS824*). For these origins we noted that the in vivo and in vitro ORC binding peaks were somewhat off set, suggesting that the binding we observed in the gEMSA might involve a site to which ORC may not normally have access in vivo. Conversely, three ARSs bound ORC more weakly than expected in the gEMSA (*ARS516*, *ARS728*, *ARS920*) based on their Kds. It is possible that some DNA fragments do not elute efficiently from the gel matrix or were underrepresented for some other technical reason. Further refinements are ongoing and will address these possibilities. Regardless, overall the gEMSA data recapitulated the origin-specific EMSA derived binding strength for 31 out of 39 origins (79.5%), indicating that the approach could be useful for identifying yeast origins that are ‘chromatin-dependent’ for ORC binding.

Comparison of ORC-DNA affinities from EMSAs with *orc2-1/ORC2* ratios of the same origins revealed a cluster of origins that relied on extrinsic (i.e. ‘chromatin’) factors for efficient ORC binding in vivo (Figure 1E). We examined whether the gEMSA recapitulated this property of these origins. The Total gEMSA signal for each origin (y-axis) was plotted for three distinct groups of origins classified by their behavior in the origin-specific EMSAs as Weak, ‘chromatin-dependent’ or DNA-dependent (Figure 2E, see also Figure 1E and associated text for origin classification information). These analyses also revealed that the gEMSA ORC-origin binding strength captured the expected differences between ‘chromatin-dependent’ and DNA-dependent ORC binding mechanisms. In particular, as predicted based on origin-specific EMSAs, putative ‘chromatin-dependent’ and Weak origins bound ORC with similar (low) ‘affinities’ in the gEMSA and were different from the gEMSA ORC-binding strengths observed for DNA-dependent origins. Therefore the gEMSA approach depicted the expected differences in ORC-DNA interactions at many different origins.

### The gEMSA showed ORC-DNA selectivity

**Figure 3:**
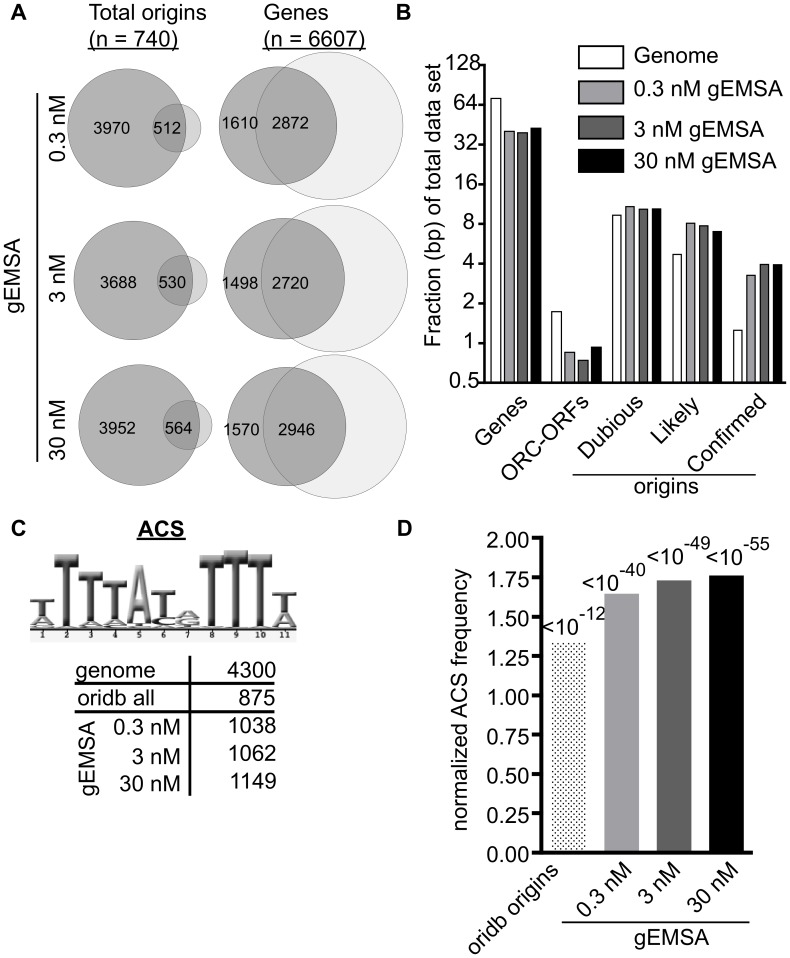
The genomic EMSA showed ORC-DNA selectivity. (A) Venn diagrams showing overlap between the complete set of annotated origins from oriDB or the complete set of yeast genes (annotated as of March 2011 on SGD) and the peaks called by ChIPotle (P value = 10^−5^) for the gEMSA data at 0.3, 3 and 30 nM ORC. (B) The fraction of base pairs in the yeast genome (y-axis) or in the gEMSA data set are shown for the genomic loci indicated on the x-axis. (C) Number of matches to the 11 bp ORC binding site consensus-motif (ACS) that were found in the indicated data sets. (D) The enrichment of an ACS motif (shown in (C)) was determined by plotting the normalized frequency (normalized to the frequency that the same motif is found within the whole genome) that an ACS match meeting a LOD cut-off of 70% was found within all of the base pairs comprising the relevant data set (x-axis). P-values for significance of enrichment indicated above bars.

The gEMSA identified a large number of ORC binding peaks at the selected stringency (P value>/ = 10^−5^) (Figure 3A and Figure S1 for maps of chromosome III and VI). This fact was not surprising given that no competitor DNA was used in the gEMSA ORC binding reactions and that the genome contains 4300 matches to the ORC binding site compared to only ∼400–700 bound by ORC in vivo. However, it was important to assess the selectivity of the gEMSA for predicted ORC binding sites. Figure 3A shows the overlap between the gEMSA peaks and either all origins (including Likely and Dubious origins) annotated in the OriDB (n = 740) or the total number of annotated yeast genes (n = 6607). A high proportion of annotated origins overlapped with the gEMSA peakes (69%, 72% and 76% for the 0.3 nM, 3 nM and 30 nM ORC arrays, respectively). While this analysis does not account for peak size or degree of overlap between peaks and origins, these data suggest that a majority of annotated origins were bound by ORC. In contrast, only 43%, 41% and 44% of annotated genes overlapped with the gEMSA peaks generated by the 0.3 nM, 3 nM and 30 nM ORC concentrations, respectively. We note that in *S. cerevisiae*, intergenic regions are small and therefore many origins are annotated as overlapping with genes simply for that reason. Furthermore, there are multiple *bona fide* ORC-binding sites within yeast genes. Thus some overlap with genes was not unexpected. Next we examined the sequence annotations overlapping the gEMSA peaks in comparison to the genome (Figure 3B). This analysis showed that while both genes and ORC-ORFs (protein coding regions that associate with ORC in vivo [51]) were depleted in the gEMSA relative to the genome, two classes of origins, likely and confirmed, were enriched. Interestingly, confirmed origins showed the greatest level of enrichment (∼4 fold over genome) suggesting that ORC bound more of these loci through specific ORC-DNA interactions compared to origins in either the dubious or likely categories. Together Figures 3A and 3B indicate that the gEMSA captured expected selectivity of yeast ORC-DNA interactions.

We reasoned that if ORC was showing DNA sequence specificity in the gEMSA data, then an optimal ORC binding site motif should be enriched regardless of whether that motif actually existed within a *bona fide* chromosomal origin because ORC was free to sample all genomic DNA in these experiments. Therefore we queried the relevant data sets for the 11 bp ACS (ARS consensus site), a conserved motif within the ORC binding site essential for origin function (Figure 3C). We also queried the gEMSA data sets for more stringently defined ORC binding sites as shown and described in Figure S2. The genome (12.1 Mbps) contained 4300 matches to the ACS while the OriDB (1.9 Mbps) contained 875 matches, a ∼1.3 fold increase in motif frequency. The ORC gEMSA datasets had a ∼1.6-fold increase in the 11 bp ACS motif frequency over the genome (1038, 1062, and 1149 matches to this ACS at 0.3 nM (1.8 Mbps), 3 nM (1.8 bps) and 30 nM (1.9 Mbps) ORC concentrations, respectively). Even greater enrichment was seen for more stringently defined matches to the ORC binding site (Figure S2). Thus as predicted if ORC-DNA sequence specificity were a greater driving force behind ORC binding in the gEMSA compared to ORC binding in vivo, DNA sequences preferred by ORC were more enriched in the gEMSA data sets than in the OriDB (Figure 3D). These data provide compelling evidence that chromatin regulates ORC-origin binding both positively and negatively.

We also analyzed the gEMSA data for the presence of motifs representing the binding sites of 89 different sequence-specific DNA binding proteins to test whether other elements were enriched by ORC, either because ORC might be capable of binding these sequences directly or because these motifs are often associated with ORC binding sites (Table S1). Motifs for only five of the 89 proteins met the initial cut-off (LOD>/ = 60%: Mata2, Nhp6a, Nhp6b, Sfl1 and Sum1), and only the Sum1 motif met a P-value cut-off of 10^−5^ at more stringent LOD scores (>/ = 80% LOD). The Sum1 motif is AT-rich as is the ORC binding site, and, perhaps relevantly, Sum1 has been implicated in origin regulation [56]–[58]. Regardless, the enrichment of origins and sequences preferred by ORC compared to other motifs indicated that the gEMSA was capturing ORC's known sequence specificity. Thus, together, the data in Figures 2 and 3 indicated that the gEMSA captured ORC's affinity and specificity for many origins.

### Features extrinsic to the ORC binding site dominated ORC-origin binding at ∼40% of yeast origins that bound ORC with high affinity in vivo

**Figure 4:**
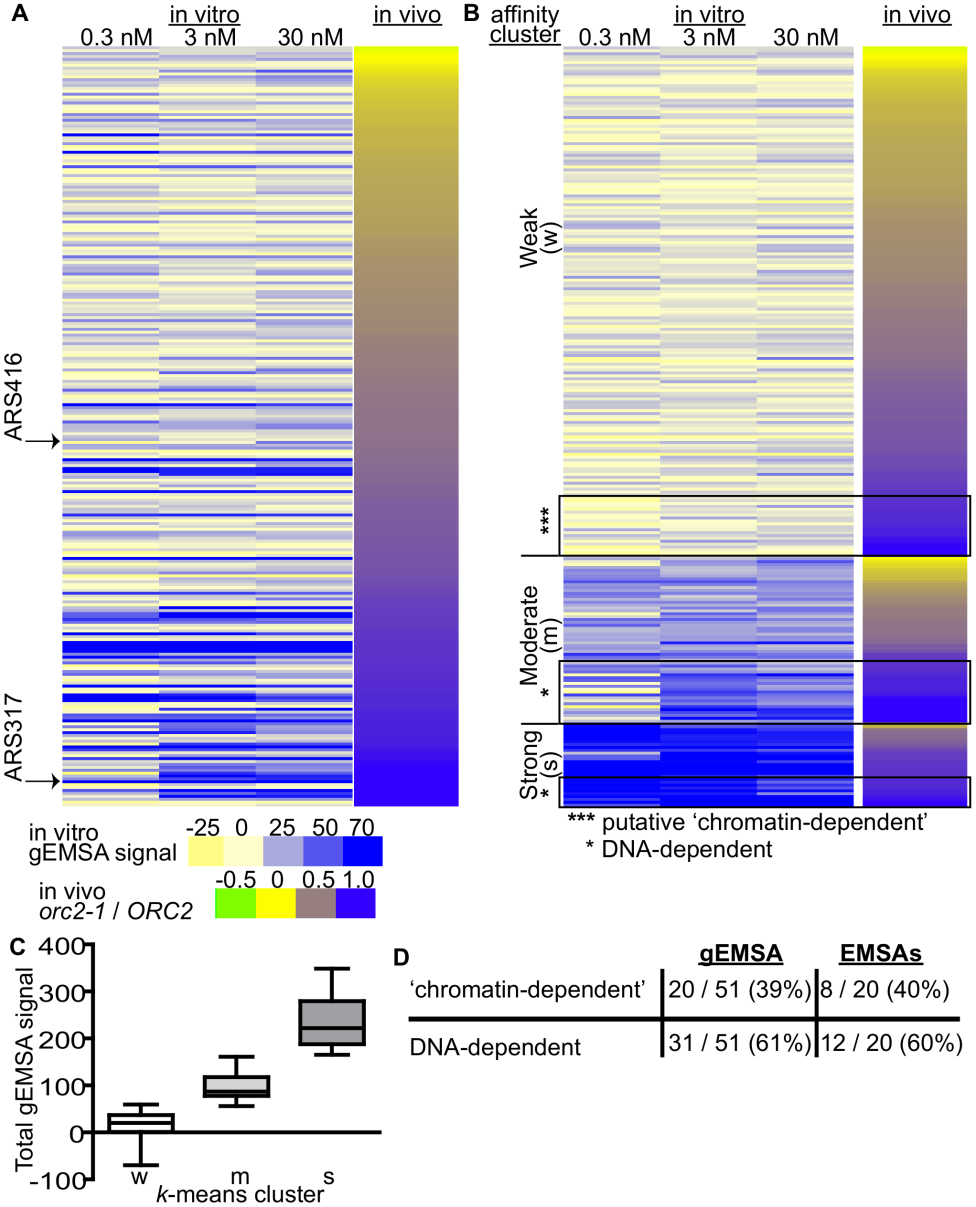
Features extrinsic to the ORC binding site dominated ORC-origin binding at many origins. (A) Heat map of peak areas overlapping the 261 confirmed origins identified in the gEMSAs. The origins are ranked by their *orc2-1/ORC2* ratios (in vivo affinity, narrow heat map). Positions of *ARS1* (*ARS416*) and *ARS317* are indicated. (B) The gEMSA data in (A) were clustered using *k*-means clustering. Within each of the three clusters, the origins were ranked by their *orc2-1/ORC2* ratios. The boxed origins in the weak cluster (W) were called ‘putative chromatin-dependent’ origins. The origins for the DNA-dependent cluster were obtained from the Moderate (M) and Strong (S) clusters (n = 31). Core-X telomeric ARSs within this group were removed prior to all subsequent computational analyses so that for the working set of DNA-dependent origins n = 20. (C) Box-and-whisker plots of the total gEMSA signal for each of the three clusters in (B). (D) Based on analyses of the gEMSA data in (C), 39% of the ‘high in vivo affinity’ origins (*orc2-1/ORC2*>/ = 0.8) were ‘chromatin-dependent’, and 61% were DNA-dependent. These percentages agreed well with those calculated from the 39 origin-specific EMSA Kds shown in Figure 1E.

Our gEMSA data provided a measure of the in vitro ORC-DNA bining strength that we could compare to the in vivo ORC-origin binding strength (*orc2-1/ORC2* ratio) from the ChIP-chip experiment. In figure 4A origins are ranked from lowest (top) to highest (bottom) in vivo affinity (i.e. *orc2-1/ORC2* ratios), and their corresponding in vitro binding strengths (i.e. gEMSA peak signals for each ORC concentration) are shown. Data for *ARS1* (a.k.a. *ARS416*) and the DNA-dependent origin *ARS317* (a.k.a. *HMR*-E silencer) are indicated. As was observed in the analysis of the 39 origins in Figure 2C, in general lower in vivo affinities (yellow; low *orc2-1/ORC2* ratios) were generally associated with weaker in vitro binding (low gEMSA signals), while higher in vivo affinities (purple; high *orc2-1/ORC2* ratios) were associated with stronger in vitro binding (stronger gEMSA signals). The co-variance of the *orc2-1/ORC2* ratios and the gEMSA data had a Pearson r coefficient of 0.20 (P-value = 0.002) indicating a positive relationship, as expected based on the proof-of-principle experiments in Figures 1 and 2 (Figure S3).

We clustered the 261 origins based on their in vitro ORC-origin binding strength into three distinct clusters: Weak, Moderate and Strong (Figure 4B and 4C). Interestingly, the majority of confirmed origins (n = 176) were in the Weak cluster. We reasoned that the origins that might rely most substantially on interactions extrinsic to ORC-DNA for ORC binding were those that bound ORC with a high affinity in vivo (purple; *orc2-1/ORC2* ratios>/ = 0.8) within the Weak in vitro (gEMSA) cluster (*** in Figure 4B). There are 51 origins with *orc2-1/ORC2* ratios>/ = 0.8, and of these 20 were found to have weak (gEMSA) in vitro ORC-DNA affinity. Importantly these twenty origins included the five previously categorized as ‘chromatin-dependent’ for ORC binding (Figure 1E), as well as one defined as ‘complex’ (*ARS1332*) (Table 2). Only one origin categorized as DNA-dependent, *ARS516*, (Figure 1E) was also found within this group. *ARS516* has a Kd of 28 nm that is close to the arbitrary cut-off used to define ‘chromatin-dependent’ origins. Thus *ARS516*'s appearance in this group was not completely surprising. Importantly, *ARS516* was the only origin classified by EMSA as DNA-dependent (Figure 1E) that fell within this putative ‘chromatin-dependent’ origin group based upon the gEMSA data. In addition, not a single origin within the EMSA-defined ‘chromatin-dependent’ group fell within the DNA-dependent group defined by the gEMSA. These observations suggest good agreement between the origin-specific EMSA and gEMSA data, as we would expect based on the analyses in Figure 2. Seven of the newly identified putative ‘chromatin-dependent’ origins had confirmed ORC binding sites, and therefore we measured ORC's interaction with these sites directly by origin-specific EMSAs (Table 2). Each of these seven origins bound ORC with Kds>4x *ARS317*, validating their placement in this ‘chromatin-dependent’ category. Thus the gEMSA approach successfully identified origins where stable binding of ORC in vivo likely relied on features extrinsic to the paradigmatic ORC-DNA interface. Based on these analyses, ORC used interactions with factors extrinsic to the established ORC binding site for stable association with up to 40% of yeast origins (Figure 4E).

**Table 2:** ‘Chromatin-dependent’ origins identified based on clustering of the gEMSA data.

origin	*orc2-1* /*ORC2*	apparent Kd (nM)	ACS status
ARS1015	0.81	63.13+/−4.3	Confirmed^1^
ARS815	0.81	Not tested	
ARS1114	0.82	33.29+/−2.4	Confirmed
ARS1307	0.82	83.7+/−3.0	Confirmed
ARS305	0.83	33.07+/−2.3	Confirmed^2^
ARS1513	0.83	56.30+/−4.7	Confirmed
ARS428	0.83	91.6+/−4.2	Confirmed
ARS447	0.84	Not tested	
ARS1116	0.84	Not tested	
ARS609	0.86	Not tested	
**ARS1332**	**0.88**	35.29+/−0.88	**Confirmed**
ARS516*	*0.88*	27.76+/−1.4	Confirmed
**ARS105**	**0.88**	>418.9	**Confirmed**
**ARS1521**	**0.94**	90.13+/−2.36	**Confirmed**
**ARS1528**	**0.95**	>418.9	**Confirmed**
**ARS1529.5**	**0.98**	191.6+/−18.98	**Confirmed**
ARS1005	1.03	>418.9	Confirmed
ARS1016	1.05	Not tested	
ARS1618.5	1.11	Not tested	
**ARS219.5**	**1.27**	>418.9	**Confirmed**

The twenty ‘chromatin-dependent’ origins identified in Figure 4C: The origins are listed top to bottom in order of their *orc2-1/ORC2* ChIP-chip peak area ratios. The six origins in bold were already classified as ‘chromatin-dependent’ based on the origin-specific EMSA data in Figure 1E and Table 1. Of the thirteen remaining origins, seven had confirmed ORC binding sites (1: [92]; 2: [92], [95], [96]). The apparent Kds for these sites were determined by ORC-EMSAs and are indicated. *ARS516* is the only DNA-dependent origin from Figure 1E that was identified by the gEMSA clustering in Figure 4C as ‘chromatin-dependent.’ The apparent Kd for *ARS516*-ORC places this *ARS* near the arbitrary ‘chromatin-dependent’ cut-off used in Figure 1E, suggesting that ORC-origin DNA and ORC-chromatin interactions both contribute to ORC binding to this origin.

### Local nucleosome architecture and its dependence on ORC around selected groups of origins

**Figure 5:**
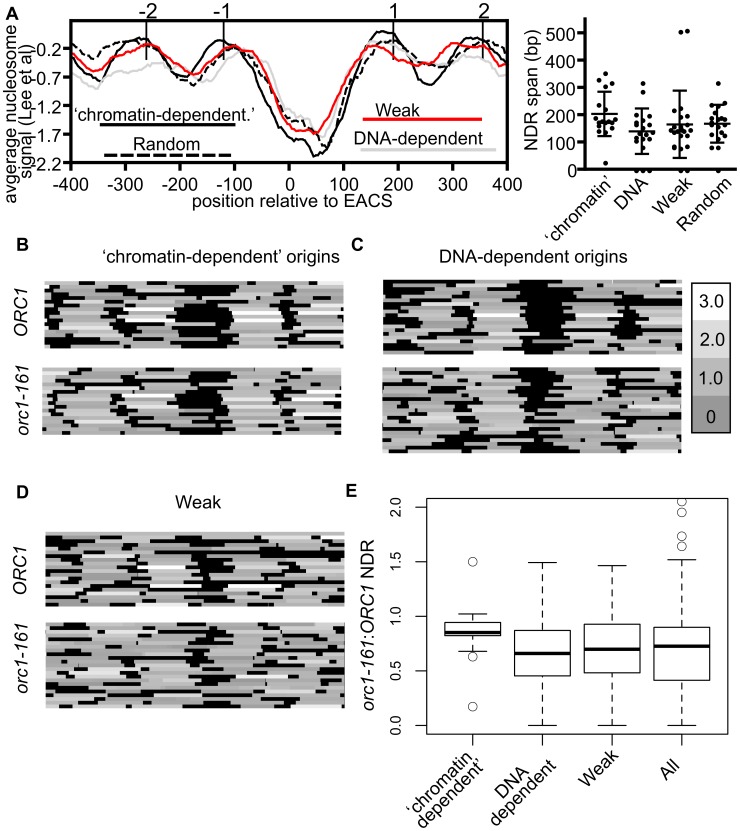
Local nucleosome architecture and its dependence on ORC around selected groups of origins. (A) Average nucleosome signals (left panel) and size of NDRs relevant to the indicated origins. Box-and-whisker plots showing median, lower and upper quartiles (box edges) and minimums and maximums excluding outliers for the NDR size of the origins are shown (right panel). These analyses used nucleosome data from [59]. (B–D) Nucleosome heat maps shown around the indicated origins in G1-phase in wild-type cells or *orc1-161* cells shifted to the non-permissive temperature for *orc1-161* [44] [63]. (B) ‘Chromatin-dependent’, (C) DNA-dependent, (D) and Weak origins. (E) Box-and-whisker plots indicating effects of removing ORC (*orc1-161*) on size of the NDR for ‘chromatin-dependent’, DNA-dependent, Weak and all 261 origins (All) examined in this study. The difference in means between the ‘chromatin-dependent’ and DNA-dependent origins had a P-value = 0.035.

The experiments discussed above defined two classes of origins that had similar affinities for ORC in vivo but had different ORC-DNA interaction strengths in vitro. To address whether local chromatin structure might be associated with these effects, we examined the average nucleosome signals relative to the ORC binding site at twenty DNA-dependent, and eighteen ‘chromatin-dependent’ origins (Figure 5A) [59]. For the DNA-dependent group, we excluded the 11 core-X telomere-associated origins for these and all subsequent analyses because they contain repetitive sequences and telomeric chromatin can affect origin function [33]. As controls we also examined 20 randomly selected origins and 20 origins from the Weak category (i.e. bound ORC poorly in vitro and in vivo). These plots revealed that each origin group was similar in having a nucleosome depleted region (NDR) 3′ of the ORC binding site, as expected. For the ‘chromatin-dependent’ origins the NDR was on average slightly larger and the flanking nucleosomes more pronounced (i.e. less ‘fuzzy’ suggesting more stable positioning) than those surrounding the DNA-dependent origins or the control groups. In addition, ‘chromatin-dependent’ and DNA-dependent origins showed differential association in terms of the gene orientation that surrounded them, consistent with local differences in chromatin environments that might result from differences in local transcription associated activities (e.g. transcription termination versus initiation) (Figure S4).

While the nucleosome positioning analyses revealed that local nucleosome positioning was, in general, similar between the origin-groups defined here, they also revealed that ‘chromatin-dependent’ origins were distinct from Orc1BAH-dependent origins defined in a previous study [43]. Specifically, Orc1BAH-dependent origins require the bromo adjacent homology (BAH) domain present on the N-terminus of Orc1 for normal levels of ORC binding in vivo [43]. The Orc1BAH domain is a nucleosome-binding module [60], [61], and origins that require the Orc1BAH domain for ORC binding have a distinctive local nucleosome architecture including a smaller NDR compared to Orc1BAH-independent origins and a striking shift of the −2 and −1 nucleosomes toward the ORC binding site [43]. Thus there were clear differences between the local nucleosome architectures of Orc1BAH-dependent origins and ‘chromatin-dependent’ origins. Consistent with these observations, ‘chromatin-dependent’ and Orc1BAH-dependent origins comprised distinct groups (Figure S5).

Both intrinsic (DNA sequence) and extrinsic factors (e.g. nucleosome remodelers, sequence-specific DNA binding proteins) help define the average nucleosome occupancy profiles that exist in vivo [62]. For example, the sequence of the ORC binding site tends to exclude nucleosomes, whereas other sequences, such as nucleosome positioning elements (NPEs) favor nucleosomes [44]. To ask whether ‘chromatin-dependent’ and DNA-dependent origins might differ in this respect, in vitro and in vivo nucleosome occupancy profiles were assessed at these origin groups and two additional groups, Weak origins and randomly selected origins (Figure S6). For ‘chromatin-dependent’ and Weak origins a difference between in vitro and in vivo nucleosome positioning for the +1 nucleosome was evident compared to DNA-dependent and randomly selected origins, which were more similar to each other. These data suggest that extrinsic factors are more relevant to positioning the +1 nucleosome, in particular, at ‘chromatin-dependent’ and Weak origins relative to DNA-dependent origins.

Experimental evidence from both in vivo and in vitro studies reveal that ORC binding to its sites within replication origins helps position neighboring nucleosomes and maintain an NDR [44]. Thus in the absence of ORC, nucleosomes normally positioned on either side of the origin encroach toward the origin reducing the size of the NDR. To determine whether ‘chromatin-dependent’ and DNA-dependent origins differed in their requirement for ORC to position flanking nucleosomes, we compared nucleosome positioning in wild-type cells to mutant cells where ORC-origin binding was abolished (*orc1-161* cells in G1 phase incubated at the non-permissive temperature) [44], [63] (Figure 5B–E). At ‘chromatin-dependent’ origins, loss of ORC binding had, on average, a comparatively small effect on local nucleosome positioning compared to all other origin groups analyzed. In particular, the positions of the −1 and +1 nucleosomes did not change substantially at many of the ‘chromatin-dependent’ origins, resulting in only modest reductions in the average size of the NDR and a tight distribution around the average (Figure 5E). In contrast, at DNA-dependent and Weak origins loss of ORC binding resulted in more dramatic changes in local nucleosome positioning, particularly the positioning of the −1 and +1 nucleosomes, resulting in a larger reduction in the size of the NDR surrounding these origins. Both of these groups behaved more similarly to all origins compared to the ‘chromatin-dependent’ group. These data suggest that ‘chromatin-dependent’ origins do not rely as heavily on ORC binding compared to other origins to establish the normal NDR.

In summary, the local nucleosome architecture of ‘chromatin-dependent’ and DNA-dependent origins, while similar, relied on different mechanisms to establish this architecture. Furthermore ‘chromatin-dependent’ origins were distinct from the previously defined group of Orc1BAH-dependent origins. These data were consistent with the hypotheses that ORC recognized a distinct chromatin-environment at ‘chromatin-dependent’ origins and that more than one type of ORC-chromatin interaction influenced origin selection by ORC in vivo. Plasmid loss assays of selected ‘chromatin-dependent’ and DNA-dependent origins suggested that the former origins were more sensitive to native, local chromatin configurations (Figure S7). This observation is consistent with the idea that local chromatin configurations reflect functional differences between ‘chromatin-dependent’ and DNA dependent origins. Therefore, while an ORC binding site motif can be found in both groups of origins (Figure S8), we found that there were differences in the local chromatin architecture. Collectively these data suggested that differences between the ORC sites and local chromatin structure were relevant to the different modes of ORC-origin binding.

### Association between ORC-origin binding mechanisms and the time of origin activation

**Figure 6:**
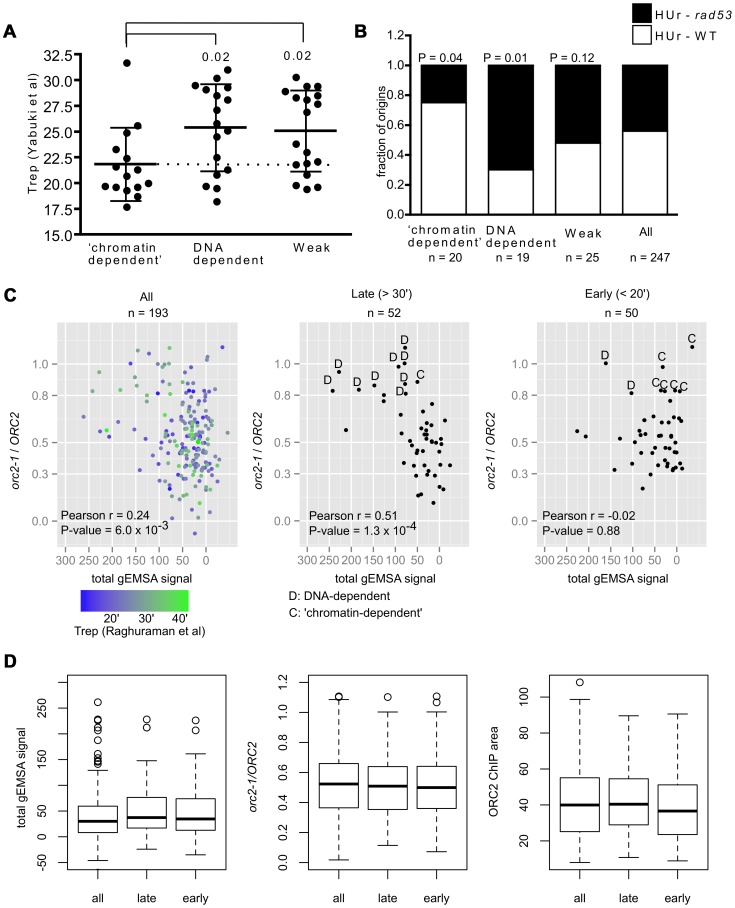
Association between ORC-origin binding mechanisms and the time of origin activation. (A) Vertical scatter plots of Trep values for three different classes of origins [64]. Dotted line extends from the mean for ‘chromatin-dependent’ origins. Similar results were observed with other data sets (Figure S9). P-values for significance of differences in the means between DNA-dependent or Weak and ‘chromatin-dependent’ origins are indicated. (B) The ability of origins in each of these groups to fire in the presence of HU in either wild-type cells (HUr-WT, white) or only in *rad53* mutant cells (HUr-*rad53-1*, black) is indicated in bar graphs. Data were from [66], [67]. P-values for the significance of the differences in distributions between selected origin groups and ‘all’ origins are indicated. (C) The *orc2-1/ORC2* ratios (y-axis, in vivo binding ORC-origin binding strength) were plotted against the Total gEMSA signal (x-axis, in vitro ORC-origin binding strength) for all origins common to this study's working data set and the Meselson-Stahl timing data set [68], for the 52 latest-firing origins (Trep>30 minutes), and the 50 earliest-firing origins (Trep<20 minutes). (D) Distributions in binding strength (gEMSA signals summed over all three concentrations of ORC), in vivo binding strength (*orc2-1/ORC2* ratio), and Wild-type (*ORC2*) ChIP peak area for all 193 origins in (C), the 52 latest origins in (C) and the 50 earliest origins in (C).

In a previous study, we showed that the high-affinity ORC-DNA interaction at *ARS317* contributed to its late-activation time and inefficiency as origin [41]. To ask whether this observation remains relevant when many origins are examined and to address whether ORC-origin binding mechanisms, as opposed to ORC-origin binding affinity *per se*, might be relevant to this origin regulation, the mean replication time (Trep values) acquired from a copy-number based analysis of DNA replication, was assessed for several groups of relevant origins [64] (Figure 6A). We observed a difference between the mean Treps for ‘chromatin-dependent’ and DNA-dependent origins. In general, this difference was observed with additional data sets that measured origin replication time directly or other properties linked to origin replication time (Figure S9). Because ‘chromatin-dependent’ ORC binding sites had weak intrinsic ORC-origin DNA interactions, one possibility was that this difference in timing in Figure 6A could be explained by weak ORC-origin binding alone. Therefore we also examined the Trep times for a comparable group of Weak origins. In contrast to ‘chromatin-dependent’ origins, origins within this Weak class were distributed over the timing spectrum and produced a mean Trep time similar to that of the DNA-dependent class of origins. We note that for origins within this Weak group, the sensitivity of our current assays did not allow us to distinguish between DNA-dependent and ‘chromatin-dependent’ ORC binding mechanisms, and thus both types of origins may be present in this Weak class. Regardless, these data indicated that enrichment for early-firing origins was a distinct property of the ‘chromatin-dependent’ group.

A replication origin's sensitivity to the ribonucleotide reductase inhibitor hydroxyurea (HU) correlates strongly with origin activation time and is often used to distinguish between origins that activate early and late in S-phase. In general early-firing origins are resistant to HU, firing efficiently even in its presence, whereas late-firing origins are sensitive to HU, failing to fire in its presence unless the Rad53-dependent checkpoint is inactivated [65]. Thus Rad53 function prevents origins that normally fire later in S-phase from activation in the presence of HU. Two genome-wide studies have measured origin activation in the presence of HU in wild-type and *rad53* mutant cells [66], [67]. We used these data to determine the origin activation behavior of our various origin groups in the presence of HU. ‘Chromatin-dependent’ and DNA-dependent origins showed different origin activation behavior in the presence of HU (Figure 6B). Specifically, fifteen of the 20 ‘chromatin-dependent’ origins were activated in the presence of HU in wild-type cells (HUr-WT) while five required inactivation of Rad53 (*rad53*) for their activation under these conditions (HUr-rad53-1). The DNA-dependent origins behaved in the opposite manner; only four of the twenty activated in the presence of HU in wild-type cells, while fifteen required inactivation of Rad53. The control group of ‘Weak’ origins distributed between the two types of activation in HU, producing a distribution similar to that observed for all origins, suggesting weak ORC-DNA interactions *per se* could not explain the skew observed for ‘chromatin-dependent’ origins. Thus based on both direct measurements of replication time during S-phase and HU-sensitivity, ‘chromatin-dependent’ origins were functionally different from DNA-dependent origins even though both groups bound ORC with similar strengths in vivo. Thus an origin's high-affinity for ORC *per se* was not strongly associated with its behaving like *ARS317* in terms of origin activation time. Rather, high-affinity binding achieved by sequence-specific ORC-origin DNA interactions was strongly associated with an origin behaving like *ARS317*.

The association between origin activation time and differences in ORC-origin binding mechanisms was striking. Because we defined in vivo binding affinity using the *orc2-1/ORC2* ratio for each origin, one concern was that the origin groups might be differentially affected by signal to noise ratios and thus that the origin activation timing effects we observed resulted from a flaw in the classification method. Therefore we performed additional control analyses (Figures S10 and S11). First, we compared the *orc2-1/ORC2* ratios of all origins compared to the *ORC2* ChIP-chip peak areas to determine whether origins with small ratios were strongly biased to small *ORC2* peak areas indicating that our ‘high-affinity in vivo’ origins might simply result from low signal to noise issues (Figure S10A). This analysis indicated that reduced *orc2-1/ORC2* ratios could be observed across the range of peak sizes and that high *orc2-1/ORC2* ratios were not clustered among the smallest peaks. Moreover, the *ORC2* peak sizes of ‘chromatin-dependent’ and DNA-dependent origins overlapped for the majority of origins in both groups. If anything, the largest skew in *ORC2* peak size toward small peaks was for the ‘Weak’ origin control group that showed no obvious skew in terms of origin activation time relative to ‘all’ origins in our data set (Figure S10B and Figures 6A and 6B). We did note however that four ‘chromatin-dependent’ origins generated *ORC2* peak areas that were smaller than any DNA-dependent origins (Figure S10A) and therefore we removed these four origins from our origin activation time analyses (Figure S11). Removal of these four smallest peaks did not affect our conclusions. These important controls provided evidence that the *orc2-1/ORC2* ratio method for classifying origins based on their relative ‘in vivo affinity’ did not bias the ‘chromatin-dependent’ origins to smaller and thus noisier peaks, suggesting that their enrichment for origins activated in early S-phase was not an artifact of the classification system.

Recent studies reveal that Forkhead transcription factors, Fkh1 and Fkh2 (Fkh1/2) regulate the origin activation time of many origins by an as yet undetermined mechanism that may involve higher-order chromosomal architecture and clustering of origins within the nucleus as well as local mechanisms [22], [26]. In particular, many early-firing origins require Fkh1/2 for their early activation time. Thus we expected that ‘chromatin-dependent’ origins, being enriched for early-firing origins, and Fkh1/2 activated origins would show some relationship, and indeed ‘chromatin-dependent’ origins were enriched for Fkh1/2 activated origins compared to DNA-dependent origins or all origins in the genome (Figure S9D) although there were early-activated origins in both groups that were distinct (Figure S12).

The analyses above revealed a striking association between the mode of ORC binding to an origin in vivo and the timing of origin activation during S-phase. However, these analyses necessarily focused on a relatively small group of origins that had a high-affinity in vivo and that we could therefore assign either DNA-dependent (n = 20) or ‘chromatin-dependent’ (n = 20) mechanisms for ORC binding. Of course many more replication origins fire either very early (Trep<20′; n = 50) or very late (Trep>30′; n = 52) in S-phase that were not represented in our groups that were formed using an arbitrary *orc2-1/ORC2* ratio cut-off of 0.8. Therefore we probed this association further by extending our analyses to origins for which Treps were determined in a genome-wide Meselson-Stahl experiment [68]. This data set was used because a larger number of origins were shared between it and the working data set in this study. All origins shared by the two data sets (n = 193), all of the latest firing (Trep>30′, n = 52) and all of the earliest firing (Trep<20′, n = 50) origins were plotted on separate graphs in which in vivo ORC-origin binding strength (*orc2-1/ORC2* ratio, y-axis) and in vitro ORC-origin binding strengths (total gEMSA signal, x-axis) were compared (Figure 6C). As expected, when all of the origins were plotted, a positive correlation was observed between in vivo and in vitro ORC-origin binding strengths (Figure 6C, left panel, r = 0.25, P = 0.006). The 52 latest-firing origins (Trep>30 minutes) showed an even greater positive relationship than all origins (r = 0.51, P = 0.0001), indicating that, as a group, these origins followed the ORC-origin DNA paradigm more closely than origins in general. In contrast, the 50 earliest firing origins (Trep<20 minutes) failed to show any relationship between in vivo and in vitro binding strength (r = −0.02, P = 0.88), indicating that, as a group, these early-firing origins did not follow the ORC-origin DNA paradigm at all. We note however that many origins among the latest and earliest firing groups lie in a similar region of the plot, suggesting that they use similar mechanisms for ORC-origin binding in vivo. Indeed, by comparing the average in vivo and in vitro affinities and ORC2 peak sizes for all of the origins examined in Figure 6C, it was evident that the latest and earliest firing origins were quite similar in terms of these values (Figure 6D). However, we note that the current resolution of the ORC-origin affinity measurements in vivo and in vitro limit our ability to definitively assign ORC-origin binding mechanisms to many of the origins that fall within a portion of the graph (*orc2-1/ORC2* ∼0.3–0.6 and total gEMSA signals <50). Nevertheless, as separate groups, the latest and earliest firing origins in the genome showed different relationships between their in vivo and in vitro binding affinities as plotted in Figure 6C. All together the data in Figure 6 provided evidence that the paradigmatic ORC-DNA interaction was a more substantial component of ORC-binding to many late-firing origins, whereas early-firing origins showed a greater dependence on ‘chromatin.’ Furthermore, the mode of ORC-origin binding (i.e. DNA-dependent versus ‘chromatin’-dependent) appeared to be more strongly associated with origin activation time than ORC-origin affinity *per se*.

## Discussion

Although the DNA replication origins of the budding yeast *Saccharomyces cerevisiae* were originally defined in part because they shared a conserved DNA sequence element, the ACS, recent work provides strong evidence that features of chromatin also contribute to defining DNA replication origins in this organism. In this study we established an unbiased, general approach for querying the relative contributions of DNA sequence versus ‘chromatin’ to origin selection by the yeast ORC. Our approach allowed for the comparison of ORC-origin interaction strengths in vivo and in vitro at a genomic scale. We then focused on origins that bound ORC with relatively high affinities in vivo where we could most confidently distinguish between the two basic binding mechanisms—sequence-specific versus ‘chromatin’-based interactions—ORC used in origin-binding. This comparative strategy allowed us to estimate ∼40% of yeast origins that bind ORC with a ‘high-affinity’ in vivo rely on features *extrinsic* to the canonical ORC-origin DNA interface for normal ORC-origin complex formation in vivo. By definition, these features are exclusive to the chromosomal context that exists in vivo on chromosomes and therefore, by the broadest definition, involve chromatin. This strategy let us examine molecular features and functional properties of ‘chromatin-’ and DNA-dependent origins, revealing unanticipated connections between distinct ORC-origin binding mechanisms and the timing of origin activation during S-phase.

### Obtaining genome-wide protein-DNA affinity measurements in vivo and in vitro

DNA sequence alone cannot explain the binding patterns of most sequence-specific DNA binding proteins in eukaryotes. Therefore a simple, quantitative approach to query how ‘chromatin’ might influence these patterns would be useful. Our approach requires that a relevant protein's ‘affinity’ for genomic DNA is measurable both in vivo and in vitro. The basic idea is that target-sites that show large discrepancies between in vitro and in vivo affinities are strong candidates for genomic loci where ‘chromatin’, as opposed to intrinsic sequence-specific protein-DNA interactions, plays a key role in the protein's binding. We exploited the established paradigm of ORC-origin DNA specificity in yeast to test this approach and distinguish between ‘chromatin-dependent’ and DNA-dependent ORC-origin binding mechanisms.

To examine ORC-origin interaction strengths in vivo we used a routine genome-wide chromatin immunoprecipitation (ChIP) approach. Because ChIP directly assesses the efficiency with which a specific DNA locus is precipitated, and many factors other than a protein's affinity for its target site can affect this efficiency, we exploited an ORC mutant (*orc2-1*) whose primary defect is to substantially reduce the concentration of ORC [52]. By comparing the efficiency of ChIP (i.e. binding signal represented as the area of a peak formed on a Nimblegen array) in *orc2-1* mutant to wild-type cells, we were able to obtain a proxy for the in vivo ‘affinity’ of most origins. However, because *orc2-1* only allowed a single low concentration of ORC to be assessed, useful information about differences in ORC-origin interaction mechanisms for origins within lower ORC-origin affinity ranges was not obtainable (e.g. the Weak class probably contains a mixture of ‘chromatin-dependent’ and DNA-dependent and complex origins). In addition, because the *orc2-1* allele destabilizes the ORC complex, sufficient amounts of the mutant complexes are not obtainable for examination of ORC-DNA interactions [69]. Thus we must also acknowledge that *orc2-1*-sensitivity of an ORC-origin interaction may reflect features of this protein-DNA complex in addition to affinity. Recent methods for reducing yeast protein concentrations in a gradual, quantized manner should be useful for improving the in vivo step of this approach [70].

To measure ORC-origin affinities in vitro we adapted the traditional gel-shift (EMSA) that is commonly used in the analysis of ORC-origin binding and that works well with many DNA binding proteins. The genomic EMSA (gEMSA) provided a simple and efficient way to isolate ORC-bound fragments away from unbound DNA, and the genomic data could be compared directly to quantitative and highly reproducible ARS-specific EMSAs. While ARS-specific EMSAs routinely include non-specific competitor, the gEMSA used the genomic DNA itself as the common source for both target sites and competitor DNA, which may be one reason a large number of binding peaks were identified. While the gEMSA did not allow us to measure actual Kd values, in general it was effective—many characterized origins with known ORC-origin DNA interaction strengths behaved as predicted in the gEMSA. Direct analyses of our group of 39 selected origins suggested that some discrepancies between in vivo and in vitro binding strengths were probably attributable to technical issues rather than biology. For example for two origins that produced strong ORC-origin binding in vitro we observed no binding in the gEMSA. Such discrepancies might result from DNA-sequence effects on chromosomal DNA shearing or recovery from DNA purification columns or the gel matrix. As in any forward genetic screen, care must be taken in evaluating positive hits. Nevertheless, based on our direct EMSAs of 39 selected origins in our initial proof-of-principle screen and an additional fourteen origins defined as either ‘chromatin-dependent’ (8) or DNA-dependent (6) using the gEMSA, we conclude that the gEMSA successfully estimated the sequence-specific ORC-origin DNA binding behavior for 44 out of 53 origins (success rate of 83%). Moreover, the functional follow-up experiments provided evidence that we had effectively distinguished between distinct classes of origins. Refinements to the approach are expected to improve our ability to classify more origins as either ‘chromatin-’ or DNA-dependent or ‘complex’, which should enable the development of more refined hypotheses about the mechanisms controlling origin selection and function in yeast.

### Flexibility in origin selection provided by a multifaceted ORC-origin interface

The Orc1BAH domain, a conserved nucleosome-binding module in Orc1 has substantial effects on ORC-origin binding in yeast and human cells [43], [46], [47]. Thus we expected that the ‘chromatin-dependent’ origins defined in this study would be enriched for origins that required the Orc1BAH domain for stable ORC binding that we defined in our previous study [43]. However, the data defied this expectation: ‘chromatin-dependent’ origins were distinct from Orc1BAH-dependent origins. This result suggests that regions of ORC, in addition to the Orc1BAH domain, contributed to ORC-chromatin interactions at origins. It also suggests that the putative Orc1BAH-nucleosome interaction does not, on its own, provide a particularly high-affinity binding interaction, a defining feature of the ‘chromatin-dependent’ origins identified in this study.

Clearly, ORC-DNA interactions also contribute to ORC-origin binding energy in yeast. Therefore, these data reveal that multiple, independent molecular interfaces contribute to ORC-origin stability. A distinct combination of molecular interfaces may define the stability and dynamics of the ORC-origin complex at any individual origin. This flexibility in the ORC-origin interface allows ORC to select origins within a wide variety of chromosomal environments even while requiring a certain level of specificity (e.g. avoiding transcribed regions). Such adaptability makes biological sense because chromatin varies substantially over a chromosome but origins must be distributed to ensure accurate duplication. While this idea has been discussed extensively with respect to metazoan origin selection, this study indicates that it is also relevant in the model *S. cerevisiae* where it can be further scrutinized at a detailed mechanistic level [71]–[73].

### Is there a conserved role for ORC-origin DNA interactions?

The defined ORC binding site was recognizable even within yeast origins where it did not appear to contribute much to ORC-origin binding energy, suggesting that ORC-origin DNA contacts play a critical role beyond stabilizing ORC's association with an origin. Indeed, several elegant biochemical studies have established that yeast origin DNA is an allosteric regulator of ORC: for example, origin DNA reduces the ATPase activity of ORC, an activity important for the MCM loading reaction in G1-phase, and ORC-origin DNA complexes stimulate Cdc6 ATPase and changes in the ORC-DNA footprint [74]–[76]. Cdc6 directly binds ORC in G1-phase, and the Cdc6 ATPase is necessary for MCM loading [75], [77], [78]. These biochemical data indicate that some features of the yeast ORC binding site likely contribute to ORC's role in loading the replicative MCM helicase complex onto origin DNA. While such a role for DNA in origin function would be expected to be fundamental and therefore conserved in other organisms, sequence-specific ORC-DNA contacts have not been observed in metazoans. It is probable, however, that an allosteric role for DNA, even for particular nucleotides, could function in the absence of any obvious sequence-specific ORC-origin DNA binding. As suggested by others, the constraints on origin function in a single-celled microbe such as yeast compared to metazoans may be an explanation for the differences in sequence-specific ORC-origin DNA interactions observed between yeast and metazoans [4]. In particular, if origin formation and function are favored within intergenic regions, yeast with their gene-rich, compact genomes offer far fewer probable positions than metazoans. This fact may increase the evolutionary pressure on yeast to establish more efficient origin selection mechanisms that include ORC-DNA specificity. In this view, metazoan and yeast origin selection are fundamentally the same; the difference is simply that because metazoan chromosomes offer so much more opportunity, the selection of any single individual origin (or MCM complex loading site) in a given region can be considerably less efficient while still supplying sufficient replicative power.

### ORC-origin interaction mechanisms and the regulation of origin activation

A particularly remarkable observation from this study was that ‘chromatin-dependent’ and DNA-dependent origins showed differential associations with origin activation time, with ‘chromatin-dependent’ origins showing greater enrichment of earlier firing, HU-resistant origins compared to DNA-dependent origins. Moreover, as a group, the latest firing origins in the yeast genome showed a positive correlation between in vivo and in vitro ORC binding affinities but the earliest firing origins did not. These data raise the possibility that ORC-origin dynamics are related in some way to mechanisms that modulate the timing of origin activation during S-phase, such as the recruitment of limiting S-phase factors that control the temporal origin activation pattern in yeast [23], [24]. One possibility is that the chromatin structures that promote early origin activation also promote ORC binding, and the weak ORC-DNA interactions that appear more enriched at earlier firing origins are a consequence of that. Alternatively, perhaps weaker ORC-origin DNA interactions associated with ‘chromatin-dependent’ ORC-origin binding have a functional purpose by promoting release of and/or conformational changes in ORC during S-phase that enhance the recruitment and/or effective function of these S-phase factors. In this view, ORC might have either a regulatory or responsive role during the S-phase portion of the ‘origin cycle’ yet to be defined [63]. Another interesting possibility is that the ORC-origin dynamics associated with early-activated origins enhances ORC's established role in loading the MCM complex onto origin DNA during G1-phase. Indeed, previous studies in both yeast and mammalian cells provide evidence that an origin's activation time is pre-determined during G1-phase, the same phase of the cell cycle when ORC and Cdc6 are loading MCM complexes onto origin DNA [79]. In addition, a relatively recent study reveals a causal relationship between ORC occupancy and MCM loading kinetics during M- and G1-phases and earlier origin activation in *S. pombe* [40], [79]. Finally, elegant biochemical studies provide evidence that cycles of ATP hydrolysis by ORC and Cdc6 that are likely coupled to ORC-origin-DNA-binding-and-release can drive reiterative MCM loading in G1-phase (i.e. multiple MCM loading events) [78], [80]. These data are consistent with a model in which the rate of an MCM loading event could be enhanced by ORC-chromatin interactions because such interactions could enhance the rate of ORC-origin-DNA-binding-and-release. Thus an origin that achieved similar levels of ORC occupancy solely through DNA contacts would be less efficient at such cycles, as ORC-origin DNA release and/or re-association would not be aided by auxiliary contacts from chromatin. This model directly connects chromatin-mediated ORC-origin dynamics to ORC's established biochemical role in loading an MCM complex onto origin DNA—that is, an origin is activated early in S-phase because it possesses a greater number of MCM complexes due to accelerated ORC-Cdc6-dependent MCM complex loading in G1-phase. An attractive feature of this idea, as discussed in the literature, is that it provides a mechanism by which to achieve a defined, quantitative difference between origins that could explain why they have differential affinities for limiting S-phase factors required for origin activation [81]–[83]. However, it is critical to acknowledge that a strong correlation between MCM complex levels and origin activation time has not been reported [22], [82], [83]. Thus while observations about ORC-origin dynamics and origin activation reported here are consistent with an MCM complex effect on origin activation, many additional experiments and multiple technical approaches will be required to address this issue definitively.

Variations in replication timing between different genomic regions are controlled primarily at the level of origin activation time and are often associated with gene expression changes that drive cell differentiation. In addition, replication-timing differences can have significant consequences on other chromosomal processes, e.g. rates of mutation and evolution [84]–[86]. These observations have spurred active investigation into the mechanisms that regulate differences in origin activation times during S-phase. Several regulators of origin firing times in yeast have been identified to date, including both local (e.g. *in*-*cis* DNA elements and/or local chromatin structure) and global (higher-order chromosomal architecture) features [22], [25], [27], [28], [35]. Examples of *in-cis* mechanisms include telomeric heterochromatin and Rpd3-mediated chromatin modifications delaying origin activation time, and centromeres advancing origin activation time. The data presented in this study suggest that the ORC binding site itself may be another *in-cis* regulator of an origin's activation time and, conversely, raise the possibility that previously defined *in-cis* regulators of origin activation time may act, at least in part, by modulating ORC-origin interaction dynamics. Recent examples of mechanisms to control origin activation timing at a more global level demonstrate a strong association between higher-order chromosome structure, as measured by chromosome capture methods, and origin activation time [14], [22]. The recent yeast studies further demonstrate a role for forkhead transcription factors (Fkh1/2) in modulating origin activation time by *in-cis* mechanisms [22], [26]. While it remains possible that these effects of Fkh1/2 perturb ORC-origin dynamics, at least at some origins, it is also possible, based on data presented both here and in the Fkh1/2 studies, that ORC-origin interaction dynamics and Fkh1/2 effects are independent phenomena associated with origin activation time. Indeed, perhaps the most critical issue is that neither of these phenomena—Fkh1/2 regulation or ORC-origin binding mechanisms—is absolute with respect to its association with origin activation time. For example, of the 100 origins that have Treps</ = 25′, 43 are not defined as Fkh1/2-regulated origins. Similarly, while we observed an enrichment of early origins within our ‘chromatin-dependent’ group, several fired later in S-phase, and conversely, a few DNA-dependent origins fired early in S-phase. If differential origin activation time simply reflects origins' adaptations to functioning in diverse chromatin environments to help insure efficient and accurate chromosomal duplication, then it is reasonable that multiple independent molecular contributions, acting in different combinations at different origins, impinge on the probability that a given origin will fire at a particular time in S-phase.

## Materials and Methods

### Electrophoretic Mobility Shift Assays

ORC was purified as described [41]. For origin-specific EMSAs (Figure 1E, Tables 1, 2 and S2), 49 bp ORC binding sites were synthesized as complementary oligos, annealed, and ligated into the KpnI and NotI sites of the pSTBlue1 vector. All ORC binding sites used in EMSAs were functionally confirmed in ARS assays. To synthesize DNA probes for EMSAs, standard PCR was performed using specific primers that annealed to regions near the multilinker cloning site pSTBlue1 with 40 uCi [^32^P]dCTP. The radiolabeled 184 bp DNA probe fragments were gel extracted from acrylamide gels before use in ORC binding reactions.

EMSAs were performed in 25 mM Tris-HCl, pH 8.0, 5 mM MgCl2, 150 mM KCl, 40 ug BSA, 5% glycerol, 5 mM DTT, 1 µg calf-thymus DNA. 2 mM ATP was present in all binding reactions except for negative control reactions. Each reaction contained 5 pM of radiolabeled DNA probe and ORC at a final concentration of 0.3, 3, 15 or 30 nM. Reactions were incubated at 25°C for 20 minutes, and ORC-Bound DNA was separated from free DNA probe on 3.5% acrylamide gel run at 200 volts for 2.5 hours at 4°C. A DNA probe with the high-affinity *ARS317* ORC binding site (apparent Kd ∼7 nM) served as an internal control in all experiments. Gels were vacuum dried and exposed to phosphor screens for at least 5 hours before imaging. Apparent Kd values were derived by fitting data to a one-site binding hyperbola and constraining the Bmax to 1 in Graphpad Prism 4.0. Each reaction was performed in triplicate and the average value and standard error for the apparent Kd that was determined is reported.

### Genomic EMSA

Chromosomal DNA was isolated from 5 ml cultures of yeast grown to saturation. DNA was purified with standard phenol/chloroform/isoamylalcohol extractions, sheared through sonication to an average size of 200 bp, and silicia membrane column-purified after excising from an agarose gel. Binding reactions between ORC and 0.5 pM genomic DNA were performed as in the EMSAs except no competitor was used. Four independent reactions per ORC titration point (0.3, 3, and 30 nM) were performed, and the contents separated by gel electrophoresis as above. Because the genomic DNA was not labeled, one lane of the gel was reserved for the binding reaction between 30 nM ORC and a radiolabeled 184 bp *ARS317* EMSA probe to mark the position of ORC-bound genomic fragments. The regions of the gel containing ORC-genomic fragment complexes corresponding in size to the ORC-*ARS317* complex were excised. Excised gel fragments from the four replicates were combined and the DNA extracted from fragments with a standard NaOAC crush/soak method to yield bound DNA. Bound DNA and total DNA (unbound) were LM-PCR amplified (Sigma Whole Genome Amplification kit) for a total of 28 cycles per sample. Samples were shipped to Nimblegen for Cy3/Cy5 labeling and hybridization upon tiled microarrays (2006-10-12_Ansari_tiling_51mer) for each ORC titration point. Three total arrays were hybridized corresponding to 0.3, 3, and 30 nM ORC binding reactions. Data from the arrays were processed as described previously [51]. The distributions of log2 ratios of bound (Cy3) to unbound DNA (Cy5) for each ORC titration point were background subtracted so that the distributions centered over 0. The most repetitive probes (1.1%) were removed from analyses. ORC binding regions (peaks) were determined with the program ChIPOTle using a window size of 240 bp, a step size of 60 bp, and a P-value of 10^−5^ [87]. The raw ORC gEMSA data has been deposited to Gene Expression Omnibus (http://www.ncbi.nlm.nih.gov/geo), accession number GSE48440.

### Bioinformatic analyses

The coordinates and sequences for the 740 confirmed, likely, and dubious origins were downloaded from the OriDB (version 2011). Gene annotations and chromosomal coordinates were downloaded from SGD. Venn diagrams were generated using the web based Venn diagram generator from the Whitehead Institute of Biomedical Research (http://jura.wi.mit.edu/bioc/tools/venn.php). We chose a P-value cutoff of 10^−5^ to maximize the number of confirmed origins identified. 4482, 4218 and 4516 peaks representing 1,847,244, 1,796,733, and 1,913,647 base pairs were called in the 0.3, 3 and 30 nM ORC arrays, respectively. The fraction of base pairs within 0.3, 3, and 30 nM ORC gEMSA peaks that overlapped the chromosomal features in Figure 3B was determined using MochiView [88].

The ACS motif reported in this study (Figure 3C) was generated with a motif finding algorithm in Mochiview. Specifically, an 11 bp motif was generated from the sequences of 67 confirmed ACS elements annotated in the OriDB using a 4th order Markov model of the full genome. To determine the number, frequency, and P-value of enrichment for the ACS elements derived from the various origin groups (yeast genome, all OriDB origins, and the gEMSA) data were scanned with 11 bp windows for sequences meeting or exceeding 70% cut-offs of the likelihood-of-discovery (LOD) value of the ACS. To determine potential enrichment of ACS matches in the gEMSA and OriDB, one-tailed Fisher's Exact tests were performed in Mochiview with frequency of ACS matches in the yeast genome serving as the control.

To convert the gEMSA binding signals into a simple representation of ORC-origin binding strength, we summed the gEMSA ORC-binding strengths obtained for each ORC concentration as described in the Figure 2 legend (this value is referred to as “Total gEMSA signal” in relevant graphs in Figure 2 and Figure 6). The gEMSA ORC-binding strength for each array (i.e. amount of ORC-origin complex formation at each concentration of ORC used) was represented as the area of the peak (peak area) that defined the ORC-origin binding complex. The peak area was defined as the sum of the signals for each feature (oligo) that comprised the defined ORC-origin binding peak, and the signal for each feature was represented as the log2 of the ratio of the experimental (gel-shifted DNA; Cy5) to total (sheared genomic DNA; Cy3) (i.e. signal = log2(Cy5/Cy3). To characterize the strengths of ORC-DNA interactions in the gEMSA, a *k*-means algorithm in the Gene Cluster 3.0 software was applied to a matrix consisting of the 0.3 nM, 3 nM, and 30 nM gEMSA signals within the coordinates of in vivo *ORC2* ChIP binding sites for 261 origins [51]. Clustering was repeated 10,000 times. ORC-DNA binding strengths in the gEMSA best fit into three distinct clusters based on the number of times the clustering was found in the 10,000 repetitions and the ability to separate moderate binding from weak ORC-DNA binding strengths.

For nucleosome mapping, nucleosome intensity signals from independent data sets were used, as indicated in Figure 5. The signals were plotted around the ORC binding sites that were defined by using the ORCACS sequence as determined by [44]. For origins lacking an ORCACS sequence, the proACS sequence, an ORC binding site derived from a phylogenetic study were used [38]. Origins lacking a predicted ACS were omitted from these analyses. All nucleosome traces were centered on the start of the EACS (Expanded ACS) on the T rich strand [49], [50]. For origins containing an ORCACS match, the first nucleotide of the ORCACS on the T rich strand marked the start of the EACS. For origins containing a proACS match, the −1 nucleotide relative to the start of the proACS on the T rich strand marked the beginning of the EACS.

ChIP-chip nucleosome intensities were processed as described previously [43], [59]. For a given origin, the nucleosome intensities were positioned relative to the start of the EACS, as above. Signal intensities with 4 base pair spacing were binned, and intensities were averaged for each bin and plotted relative the start of the EACS. The size in base pairs of the nucleosome depleted region (NDR) was calculated with a Perl script, where the NDR was defined by width in base pairs between the beginning coordinate of the +1 nucleosome the end coordinate of the −1 nucleosome. The genome-wide normalized nucleosome signals mapped in vivo (growth in permissive YPD) and in vitro were positioned relative to the T rich starts of the EACSs, as above [62]. Data were smoothed by binning values into 4 base pair spacing. Bins were averaged and plotted relative to the EACS.

To determine the effect of the *orc1-161* mutation on nucleosome configuration around ‘chromatin-dependent’ and DNA-dependent origins, data from an experiment in which nucleosomes were mapped in wild-type and *orc1-161* mutant cells arrested in G1 phase at non-permissive temperatures for *orc1-161* were used [44]. Nucleosome signals were oriented in relation to the start of the T rich strand of the ORC binding sites, as described above. Nucleosome signals were plotted in heat maps. The NDRs of origins in the *orc1-161* and *ORC1* backgrounds were calculated with the Perl script as described [43].

To examine the relationship between ORC binding mechanisms and origin activation timing, several independent data sets were used. The Trep values were obtained from published data sets and downloaded from OriDB [64], [68]. T_1/2_ values were obtained from [30], [83]. To determine whether origin activation in the presence of hydroxyurea required Rad53, genome-wide data from wild-type cells and *rad53-1* mutant cells from two independent studies were combined [66], [67]. If an origin was shown to fire in the presence of HU (HU-resistant) in wild-type cells in either of these two studies, we considered it HU-resistant (HUr-WT (Figure 6B)). If it only fired in the presence of HU in *rad53* mutant cells in one or both studies, it was termed HU-sensitive (referred to in Figure 6B as HUr-*rad53*). Data were available for all 20 ‘chromatin-dependent’ origins (n = 20) but only 19 of the DNA-dependent origins (i.e. one DNA-dependent origin, *ARS1108*, was not detected under either condition in ether data set). Data for all 25 origins in the Weak class were available. To determine the association with *CLB5*-regulation (Figure S5B) and *FKH*-mediated regulation (Figure S5C and Figure S6), data from two independent genome-wide studies were used [22], [30].

### Plasmid replication assays (ARS assays)

Core origin and expanded origin constructs were PCR-amplified and cloned into the NotI site of the pARS/PM vector [43], [89]. Core origins were comprised of the DNA sequences annotated in oriDB. Expanded origins contained ∼1 kb chromosomal sequence centered surrounding a functionally confirmed ORC binding site (Table S3). The sub-clones of *ARS1529.5* in Figure S7 were generated using sequence from the *KANMX* coding sequence as substitutes for regions of *ARS1529.5* as indicated in Figure S7 and Table S3. Plasmids were transformed into wild-type W303 yeast using standard techniques, and ARS assays were performed with four independent cultures per plasmid as in [50].

## Supporting Information

Figure S1 MochiView Plots of (A) chromosomes III and (B) VI for the gEMSA data at all three ORC concentrations and for the *ORC2* ChIP-chip data are shown. Positions of ACS matches and origins called in oriDB (confirmed, likely and dubious) are also shown.
(TIF)

Figure S2 Enrichment of more stringently defined ORC binding sequence elements in the gEMSA data set. To further query how well the gEMSA captured yeast ORC DNA binding specificity, more stringently derived sequence elements were examined and compared for their enrichment in the oriDB (i.e. all sites bound by ORC in vivo, including Likely and Dubious origins) and at the three concentrations of ORC used in the gEMSA. (A) The EACS, EACS+WTW and ‘ORCACS’ motifs are shown. All three of these motifs were defined in previous studies. The EACS motif was as annotated in [48]. The EACS+WTW motif is a motif consisting of the currently defined elements comprising the bipartite ORC binding site, the EACS and the WTW of the B1 element, separated by 13 degenerate nucleotides [48], [50]. The ‘ORCACS’ motif was derived by running the Mochiview motif finding algorithm on the ORCACS sequences annotated in [44]. To the right of the motifs, a histogram graph depicting the frequency enrichment of the indicated motifs relative to the frequency the motif is found in the genome. Greater fold enrichment is observed for sequences of increasing complexity that help define the ORC binding site. P-values for significance of enrichment are indicated above bars. Frequencies were determined using Mochiview using a 60% LOD cutoff. With increasing motif complexity, or greater approximations to the actual sequence that ORC contacts, motif frequencies increase in both the complete OriDB sequences and the gEMSA sequences. The level of specificity observed in the gEMSA allowed for discerning differences in motifs with different approximations of the ORC binding site. For each of the three motifs examined, the motif frequency in the gEMSA ORC binding sites were similar to the sequences within origins annotated in the OriDB. That these motifs did not have higher frequencies in the gEMSA is not unexpected considering that these motifs were not generated with origins that had been parsed based on binding strength. (B) ORC-DNA interactions in the gEMSA can differentiate between ORC-DNA binding motifs with different ORC-DNA affinities. A weak ORC binding site (OBS) and a tight OBS consensus motif were generated from ORC binding sites from either 11 of the tightest *orc2-1*r (Tight OBS) or 11 of the weakest *orc2*-1s origins in Figure 1 (Weak OBS). The relative frequency enrichments of these motifs relative to their frequency in the genome were determined for the datasets indicated as in (A). At each ORC concentration in the gEMSA, the Tight OBS had a higher normalized frequency compared to that of the Weak OBS, as would be expected if the gEMSA were measuring ORC-DNA affinity. The gEMSA had a greater differential in Weak and Tight frequencies compared to that observed in the complete OriDB. The gEMSA was better able to distinguish between high affinity and weak affinity motifs as would be expected if the gEMSA measured ORC-DNA interactions and all genomic DNA was made accessible.
(TIF)

Figure S3 The Total gEMSA signal as a representation of ORC-origin binding strength in vitro was for each of the 261 origins that comprise the working data set for this study was plotted (x-axis) against (y-axis) (A) the *orc2-1/ORC2* ratio for each of the *ORC2* ChIP peaks called in the original ChIP-chip data set by ChIPOTle at P-value cut-off </ = 10^−20^ or (B) the area of the *ORC2* ChIP peaks called in the original ChIP-chip data set by ChIPOTle at P-value cut-off </ = 10^−20^ on the y-axis. The x-axis values were plotted right to left, from smallest to largest (weaker to strongest binding, right to left) so that the visual output is comparable to the graphs in Figures 1A and 1E, respectively, where apparent Kd is plotted on the x-axis (Kd values are inversely proportional to binding strength). Note however that while the gEMSA signal may be related to Kd it is not equivalent to this value (Figure 2).
(TIF)

Figure S4 Gene-orientation landscape surrounding ‘chromatin-dependent’ and DNA-dependent origins. (A) The orientations of genes flanking ‘chromatin-dependent’, DNA-dependent and ‘all’ origins examined in this study. ‘All’ refers to all the *ORC2* ChIP confirmed origins with peaks called at P-value 10^−20^ by ChIPOTle [51]. The groups were compared to ascertain whether one group preferentially associates with a particular type of gene orientation with respect to transcriptional initiation or termination. Convergent and divergent genes occupy opposite strands of the chromosome and have transcripts either moving towards or away from the origin, respectively and the origin is flanked on either side by termination events or initiation events, respectively. Tandem genes occupy the same strand, and thus the origin is flanked on one side by a termination event and on the other by an initiation event. ‘Other’ refers origins whose location has not been definitively assigned. P-values for significance of enrichment of any gene orientation relative to ‘all’ origins are indicated within the relevant portions of the stacked histogram. (B) ‘Chromatin-dependent’ and DNA-dependent origins were associated with different aspects of transcription relative to the orientation of their ORC binding sites. ORC binding sites were assigned as described for Figure 5. Not all origins had sequences that matched the criteria for ORC binding sites. These origins were not included in these analyses, explaining the discrepancies in ‘n’ in (A) and (B). We compared the fraction of ORC binding sites in ‘chromatin-dependent’ and DNA-dependent origins that had either a transcriptional termination event or initiation event upstream of the start of the ORC binding site. Gene orientations were compared relative to the location of the T rich strand of the ORC binding site (EACS), as in Figure S2. P-values for significance of enrichment of any gene orientation relative to ‘all’ origins are indicated within the relevant portions of the stacked histogram.
(TIF)

Figure S5 Origins requiring the Orc1BAH domain for ORC binding were distinct from ‘chromatin-dependent’ origins. In a previous study we defined a group of 34 origins that required the Orc1BAH domain for efficient binding by ORC in vivo (*orc1bahΔ/ORC1* ratio</ = 0.3) [43]. (A) Venn diagrams indicating degree of overlap between the indicated origin groups. (B) Nucleosome configuration around the indicated groups of origins relative to the ORC binding site (EACS), as in Figure 5.
(TIF)

Figure S6 Comparison of nucleosome positioning in vitro and in vivo around the indicated origin groups; these analyses used nucleosome data from [62].
(TIF)

Figure S7 Functionality of ‘chromatin-dependent’ origins was enhanced by native sequences flanking the core origin, whereas DNA-dependent origins were comparably unaffected by such sequences. ‘Chromatin-dependent’ and DNA-dependent origins were distinguished based on the mechanisms that determined their ORC-origin interactions in vivo. While both groups of origins showed similar though not identical matches to the consensus ORC binding site (Figure S8), indicating that the basic ORC binding motif was maintained, they showed different local chromatin configurations that might be functionally relevant (Figure 5). To address whether these different binding mechanisms were associated with functional differences between these two classes of origins, we examined the ability of several origins from each group to replicate a plasmid. In particular, we reasoned that if ‘chromatin-dependent’ origins depended on a distinct local chromatin environment then they might be more sensitive to the presence of additional chromosomal sequences beyond the defined ‘core’ ARS compared to DNA-dependent origins. (A) To test this idea, the origin activity of several ‘chromatin-dependent’ and DNA dependent origins was compared using ARS assays. In one set of experiments, we assessed the activity of ‘core’ origins as defined by oriDB (Table S3). Given the arbitrary fragments used to define most ARSs, ‘core’ origins vary substantially in length. Therefore selection of origins for these analyses was in part based on their annotated lengths such that the mean core size for the ‘chromatin-dependent’ and DNA-dependent groups of origins did not differ substantially (‘chromatin-dependent’ core ARSs were 279+/−33 bp while DNA-dependent core ARSs were 293+/−43 bp). In the second set of experiments, the ARS activity of the ‘expanded origins’ was assessed. An expanded origin contained the core origin plus an additional 500 bp of flanking chromosomal sequence on either side of the ORC binding site. (B) On average, ‘chromatin-dependent’ ‘core’ origins showed weaker ARS activity than DNA-dependent ‘core’ origins. However, the expanded versions of ‘chromatin-dependent’ and DNA-dependent origins exhibited similar ARS activities because the additional chromosomal sequence improved the ARS activity of ‘chromatin-dependent’ origins significantly but had little effect on the ARS activity of DNA-dependent origins. These data suggested functional differences between ‘chromatin-dependent’ and DNA-dependent origins that were consistent with the differences in ORC binding mechanisms deduced from the genomic analyses. (C) To test whether the ARS assay could be used to dissect elements that might contribute to the distinct mechanistic requirements of ‘chromatin-dependent’ origins, a specific ‘chromatin-dependent’ origin, *ARS1529.5* was examined in more detail. First, one possible explanation by which additional chromosomal sequences improved ARS activity of ‘chromatin-dependent’ origins was that these additional sequences contained independent ARS elements. However, a mutation in the confirmed ORC binding site within *ARS1529.5* abolished ARS function from the expanded form of this origin (Table S3). Thus the additional functions provided by the extra chromosomal sequences in the expanded version of *ARS1529.5* depended entirely on the confirmed ORC binding site of *ARS1529.5*, indicating that there were no independent functional ORC binding sites within these sequences. A second potential explanation was that additional sequence (i.e. larger plasmid) might improve ARS function in general [54]. Therefore to test the specificity of the additional sequences to the ARS activity of the expanded version of *ARS1529.5*, the native chromosomal sequences were swapped with *KANMX* sequences. This version of *ARS1529.5* had ARS activity as weak as the ‘core’ version of *ARS1529.5*, indicating that the native chromosomal sequences provided specific contributions that enhanced *ARS1529.5* function. The nucleosome analyses presented in Figure 5 suggested that ‘chromatin-dependent’ origins contained a distinctively positioned nucleosome on the 3′ side of the ARS, relative to the T-rich strand of the ORC binding site. To test whether the 5′ or 3′ chromosomal regions contributed differently to the enhanced ARS function of *ARS1529.5*, they were replaced individually with an equivalently sized *KANMX* region. The additional 3′ chromosomal sequences provided for enhanced ARS activity of *ARS1529.5*, creating loss rates equivalent to those produced by the fully expanded version of this ARS, whereas the additional 5′ chromosomal sequences had no ability to enhance ARS activity of *ARS1529.5*. These data suggested that ‘chromatin-dependent’ origins were sensitive to specific features in local ‘chromatin’ structure.
(TIF)

Figure S8 The consensus ORC binding site motifs derived from analyses of different origin groups. A consensus ORC binding site, including the 17 bp EACS, the WTW motif and the linker region in between these two elements was determined for ‘chromatin-dependent’ and DNA-dependent origins using Weblogo. These were compared to the consensus derived from 68 confirmed ORC binding sites (annotated and referenced on OriDB). The ORC binding sites were identified for 18 ‘chromatin-dependent’ origins and 20 DNA-dependent origins based on one of the following: a confirmed ACS, a match to an ORCACS, or a match to a proACS [38], [44], as in Figure 5. When necessary, annotated sequences were converted to a 34 bp binding site by including nucleotides 5′ and 3′ of the annotated ACS match. Sequences of ORC binding sites were aligned based on the start of the EACS on the T rich strand.
(TIF)

Figure S9 Association of ‘chromatin-dependent’, DNA-dependent and ‘Weak’ origins with various timing or timing-associated measurements. Defined in text, with: (A) Trep values determined from a Meselson-Stahl experiment [68] (B) t_1/2_ values described derived using an independent timing data set from a Meselson-Stahl experiment [29], [30], [83]. t_1/2_ is the median time of origin firing. (C) *CLB5*-dependent regulation (CDR) (Clb5 is the S-phase cyclin required for the activation of late-firing origins [29]. P-values from hypergeometric distribution function indicated. In general early firing origins are activated independently of Clb5). (D) Type of regulation by the Fkh1 and 2 (FKH) transcription factors [22].
(TIF)

Figure S10 Chromatin-dependent origins are comprised of origins associated with virtually the full range of *ORC* peak sizes in the *ORC2*-ChIP-chip. A ‘chromatin-dependent’ origin was defined based on having a high *orc2-1/ORC2* ratio binding peak ratio in ChIP (>/ = 0.8; *orc2-1*-resistant) but weak in vitro binding in the gEMSA (or, in some cases also a high Kd determined in EMSAs). A concern was that many such origins might have small *ORC2* peaks and thus *orc2-1*-resistance might result from noise in the data. These control analyses suggest that was not a major concern. (A) A plot of the log2(*orc2-1/ORC2*) (y-axis) versus log2 of *ORC2* peak area (x-axis) shows that the majority of ‘chromatin-dependent’ (blue) and DNA-dependent origins (black) corresponded to origins that generated similarly-sized *ORC2* ChIP peak areas. *Orc2-1*-resistant origins and *orc2*-1-sensitive origins corresponded to origins that generated *ORC2* ChIP peak areas across the entire range of peak areas measured. (B) Box-and-Whiskers plot of *ORC2* ChIP peak areas for the indicated origin groups. The Box-and-Whiskers plots are as described in Figure 5.
(TIF)

Figure S11 Timing and HU-resistance differences between ‘chromatin-dependent’ and DNA-dependent origins determined after removal of the four ‘chromatin-dependent’ origins associated with the smallest *ORC2* peak areas. (A) This plot is analogous to the one shown in Figure 6A of the main text. Two of ‘chromatin-dependent’ origins removed prior to these analyses had early Treps, but two were not assigned Trep values. Hence removal of all four of these origins had a negligible effect on the average Trep differences between ‘chromatin-dependent’ and DNA-dependent origins. (B) This plot is analogous to the one shown in Figure 6B of the main text. There was no substantial change in the difference between ‘chromatin-dependent’ and DNA-dependent origins in terms of HU-resistant activation after removal of the four smallest ‘chromatin-dependent’ origins. P-values indicating the significance of the differences in distribution of origin types in each group relative to all origins indicated.
(TIF)

Figure S12 Analyses of the relationship between FKH regulation and origin-binding mechanisms by ORC. Overlap among key groups (Venn diagrams) of origins is shown for: Early origins Trep (Trep<25′) that were also identified in our ORC ChIP study (n = 100) [68]; Fkh1/2 activated origins (n = 82) (57 origins with Trep<25′ were Fkh1/2 activated) [22]; ‘chromatin-dependent’ origins (n = 20); and DNA-dependent origins (n = 20). Of the 10 ‘chromatin-dependent’ origins that are fire early in S-phase, six of these are Fkh-activated, while four were not regulated by Fkh1/2 were not regulated. 43 origins out of the 100 that fire early (Trep<25′) were not called as Fkh-activated.
(TIF)

Table S1 Transcription factor DNA binding site motifs within the genomic fragments enriched in the gEMSA. 89 motifs identified in a previous protein-binding microarray experiment were analyzed for enrichment in the gEMSA data at a P value = 10^−5^ cut-off in gEMSA [90]. This table lists these motifs, the relevant Likelyhood of Discovery (LOD) cut-offs used and the P-values from Fisher Exact T tests. The conclusions are summarized in the results section of the text pertaining to Figure 3.
(XLS)

Table S2 Yeast origins analyzed in this study. Origins within the three groups—‘chromatin-dependent’, DNA-dependent and ‘Weak’—used for more detailed analyses are listed.
(XLS)

Table S3 DNA sequences used for functional ARS assays in Figure S7.
(XLS)
