# Supplementary figures and images for: A Link between ORC-Origin Binding Mechanisms and Origin Activation Time Revealed in Budding Yeast
Source: PLoS Genet. 2013 Sep 12;9(9):e1003798. doi: 10.1371/journal.pgen.1003798 (PMC3772097; doi:10.1371/journal.pgen.1003798)

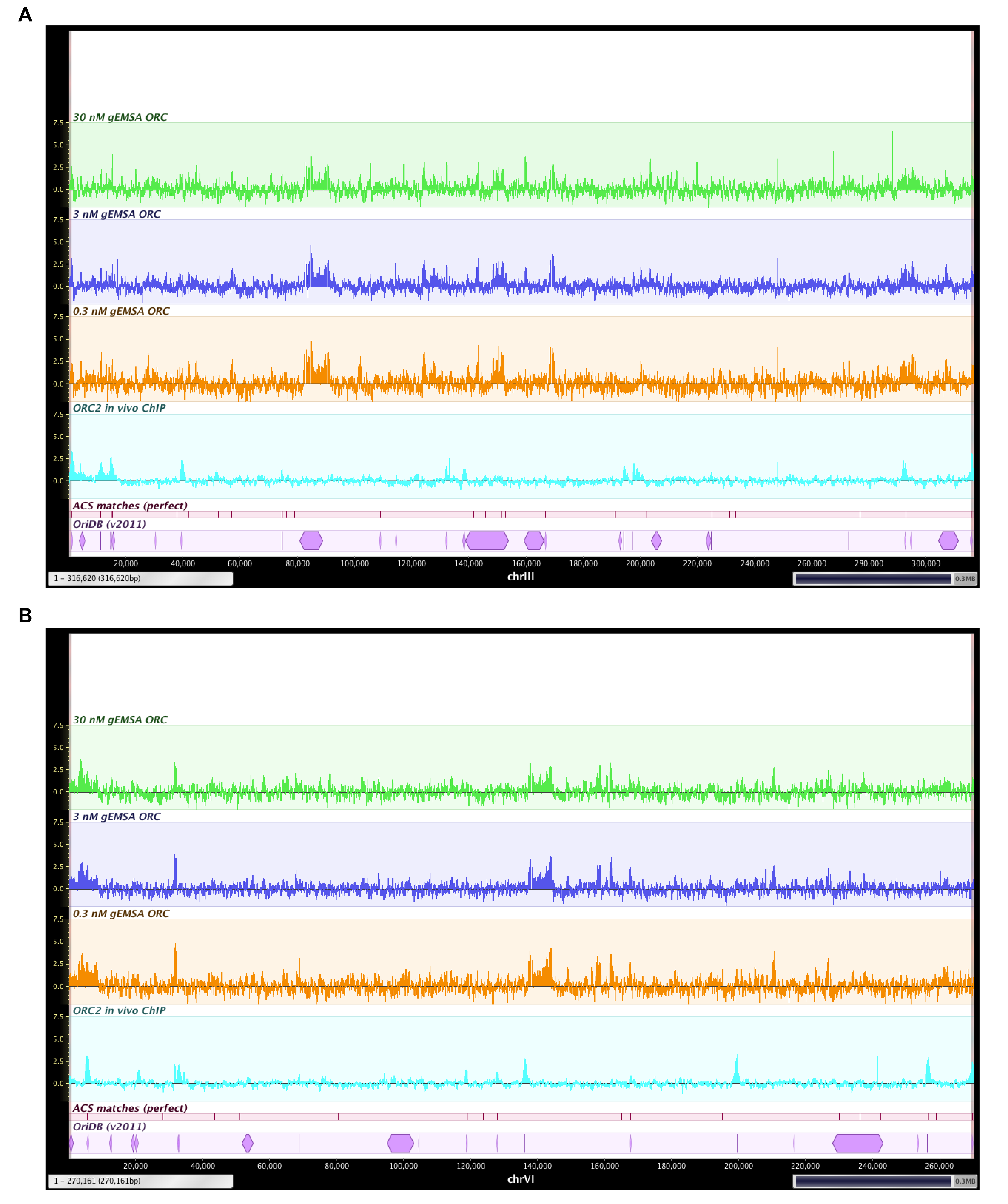

Supplement: Figure S1 — MochiView Plots of (A) chromosomes III and (B) VI for the gEMSA data at all three ORC concentrations and for the ORC2 ChIP-chip data are shown. Positions of ACS matches and origins called in oriDB (confirmed, likely and dubious) are also shown. (TIF) [file pgen.1003798.s001.tif]

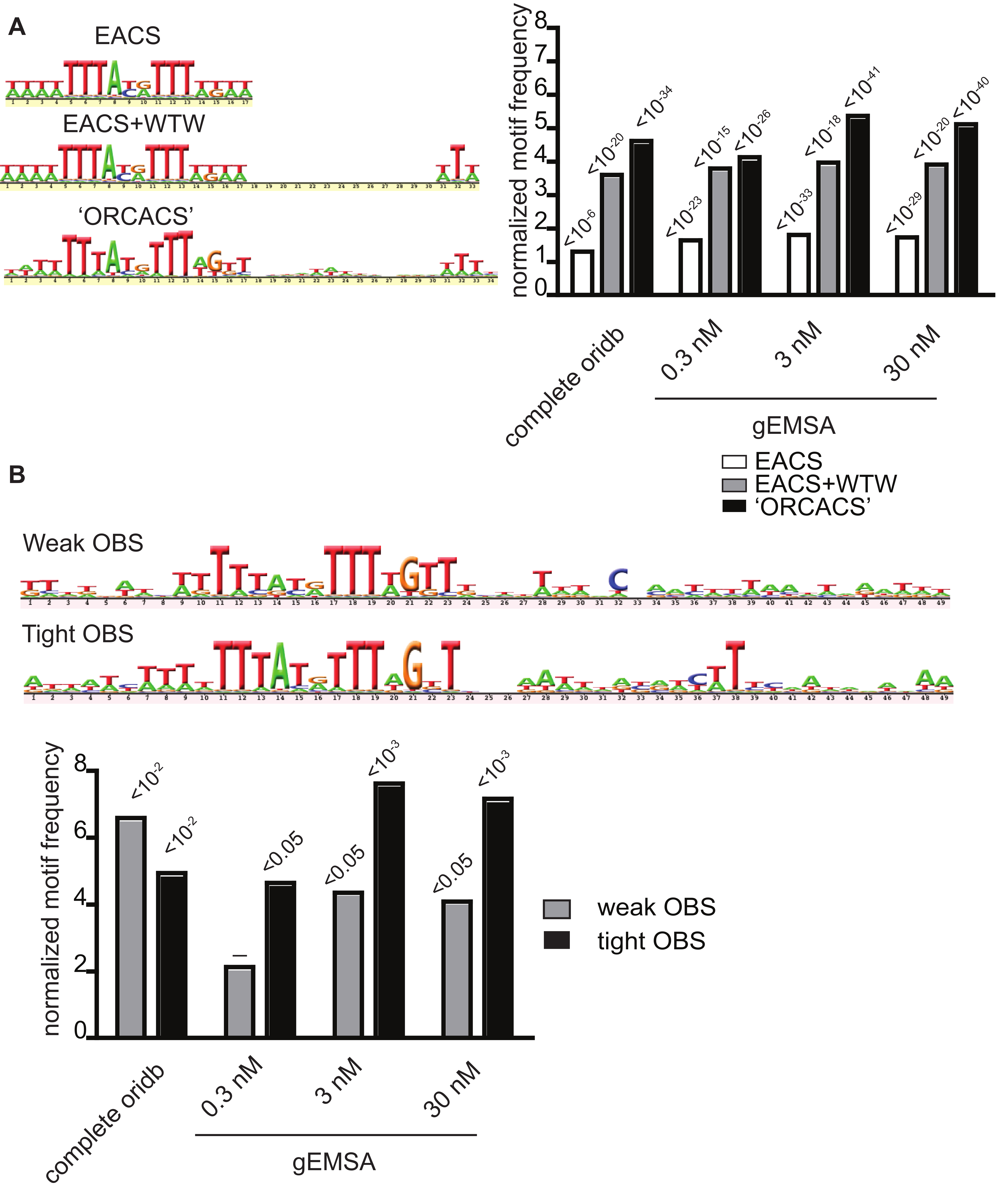

Supplement: Figure S2 — Enrichment of more stringently defined ORC binding sequence elements in the gEMSA data set. To further query how well the gEMSA captured yeast ORC DNA binding specificity, more stringently derived sequence elements were examined and compared for their enrichment in the oriDB (i.e. all sites bound by ORC in vivo, including Likely and Dubious origins) and at the three concentrations of ORC used in the gEMSA. (A) The EACS, EACS+WTW and ‘ORCACS’ motifs are shown. All three of these motifs were defined in previous studies. The EACS motif was as annotated in [48]. The EACS+WTW motif is a motif consisting of the currently defined elements comprising the bipartite ORC binding site, the EACS and the WTW of the B1 element, separated by 13 degenerate nucleotides [48], [50]. The ‘ORCACS’ motif was derived by running the Mochiview motif finding algorithm on the ORCACS sequences annotated in [44]. To the right of the motifs, a histogram graph depicting the frequency enrichment of the indicated motifs relative to the frequency the motif is found in the genome. Greater fold enrichment is observed for sequences of increasing complexity that help define the ORC binding site. P-values for significance of enrichment are indicated above bars. Frequencies were determined using Mochiview using a 60% LOD cutoff. With increasing motif complexity, or greater approximations to the actual sequence that ORC contacts, motif frequencies increase in both the complete OriDB sequences and the gEMSA sequences. The level of specificity observed in the gEMSA allowed for discerning differences in motifs with different approximations of the ORC binding site. For each of the three motifs examined, the motif frequency in the gEMSA ORC binding sites were similar to the sequences within origins annotated in the OriDB. That these motifs did not have higher frequencies in the gEMSA is not unexpected considering that these motifs were not generated with origins that had been parsed based on binding strength. (B [file pgen.1003798.s002.tif]

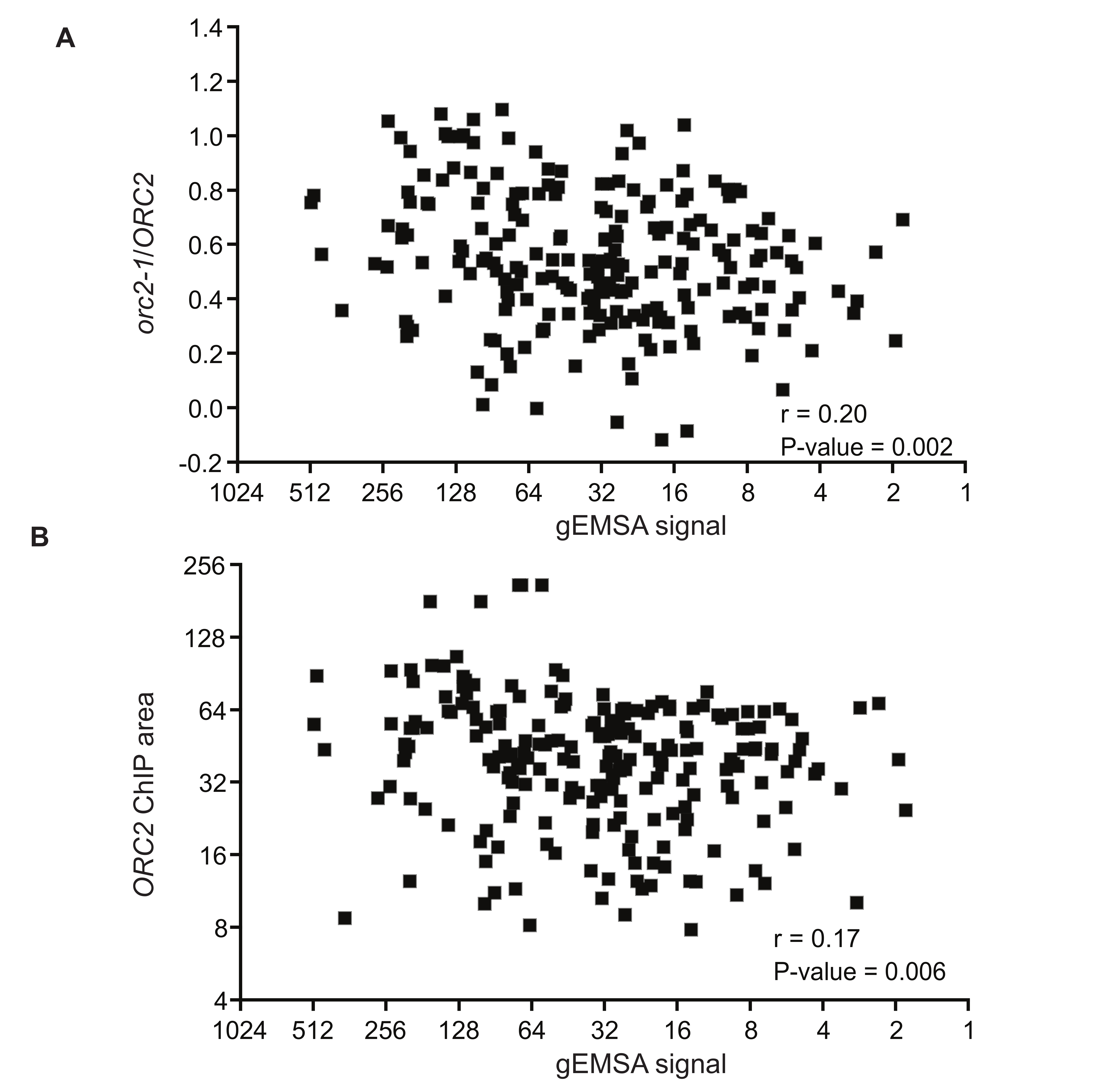

Supplement: Figure S3 — The Total gEMSA signal as a representation of ORC-origin binding strength in vitro was for each of the 261 origins that comprise the working data set for this study was plotted (x-axis) against (y-axis) (A) the orc2-1/ORC2 ratio for each of the ORC2 ChIP peaks called in the original ChIP-chip data set by ChIPOTle at P-value cut-off </ = 10−20 or (B) the area of the ORC2 ChIP peaks called in the original ChIP-chip data set by ChIPOTle at P-value cut-off </ = 10−20 on the y-axis. The x-axis values were plotted right to left, from smallest to largest (weaker to strongest binding, right to left) so that the visual output is comparable to the graphs in Figures 1A and 1E, respectively, where apparent Kd is plotted on the x-axis (Kd values are inversely proportional to binding strength). Note however that while the gEMSA signal may be related to Kd it is not equivalent to this value (Figure 2). (TIF) [file pgen.1003798.s003.tif]

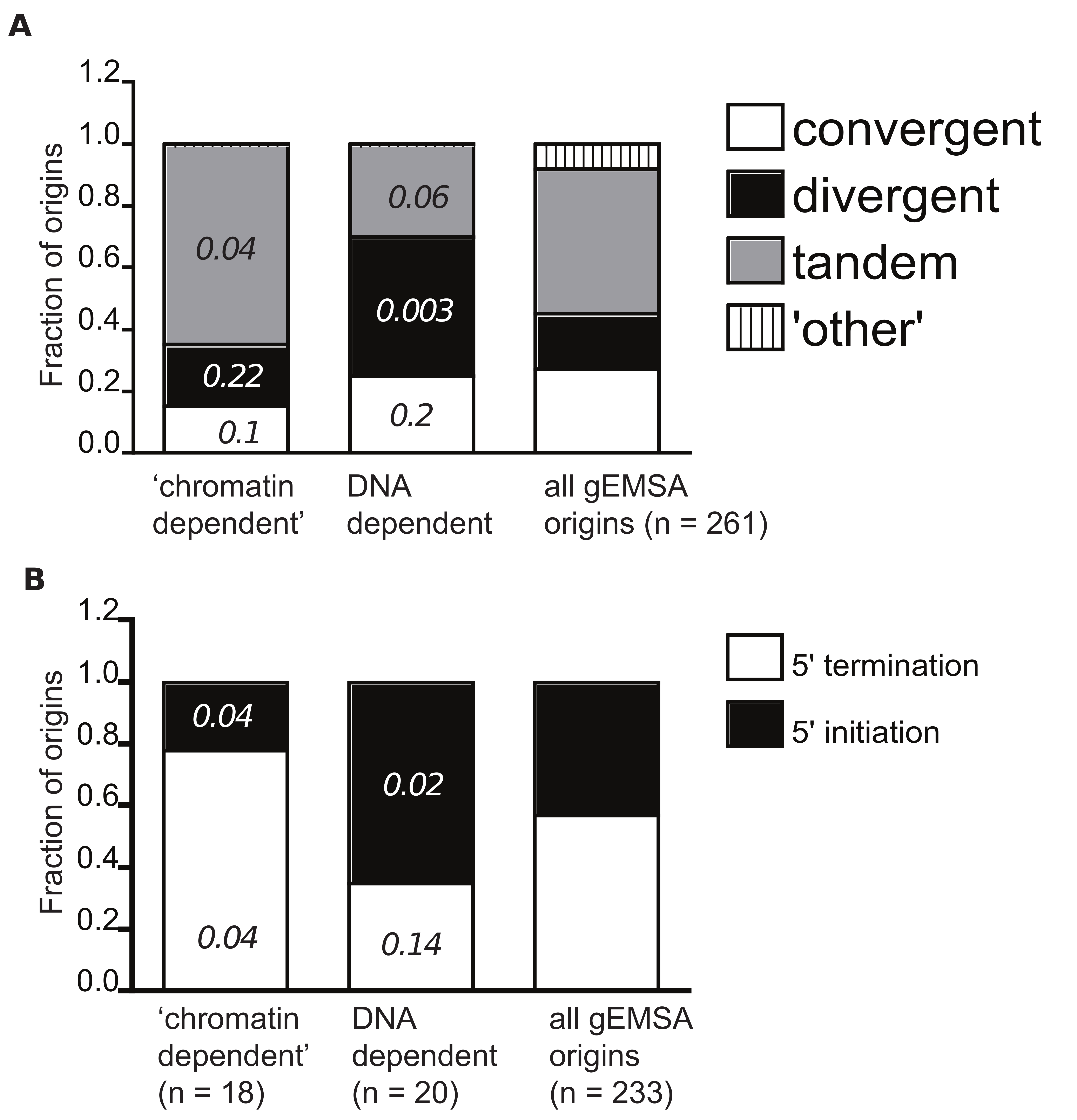

Supplement: Figure S4 — Gene-orientation landscape surrounding ‘chromatin-dependent’ and DNA-dependent origins. (A) The orientations of genes flanking ‘chromatin-dependent’, DNA-dependent and ‘all’ origins examined in this study. ‘All’ refers to all the ORC2 ChIP confirmed origins with peaks called at P-value 10−20 by ChIPOTle [51]. The groups were compared to ascertain whether one group preferentially associates with a particular type of gene orientation with respect to transcriptional initiation or termination. Convergent and divergent genes occupy opposite strands of the chromosome and have transcripts either moving towards or away from the origin, respectively and the origin is flanked on either side by termination events or initiation events, respectively. Tandem genes occupy the same strand, and thus the origin is flanked on one side by a termination event and on the other by an initiation event. ‘Other’ refers origins whose location has not been definitively assigned. P-values for significance of enrichment of any gene orientation relative to ‘all’ origins are indicated within the relevant portions of the stacked histogram. (B) ‘Chromatin-dependent’ and DNA-dependent origins were associated with different aspects of transcription relative to the orientation of their ORC binding sites. ORC binding sites were assigned as described for Figure 5. Not all origins had sequences that matched the criteria for ORC binding sites. These origins were not included in these analyses, explaining the discrepancies in ‘n’ in (A) and (B). We compared the fraction of ORC binding sites in ‘chromatin-dependent’ and DNA-dependent origins that had either a transcriptional termination event or initiation event upstream of the start of the ORC binding site. Gene orientations were compared relative to the location of the T rich strand of the ORC binding site (EACS), as in Figure S2. P-values for significance of enrichment of any gene orientation relative to ‘all’ origins are indicated within the relevant por [file pgen.1003798.s004.tif]

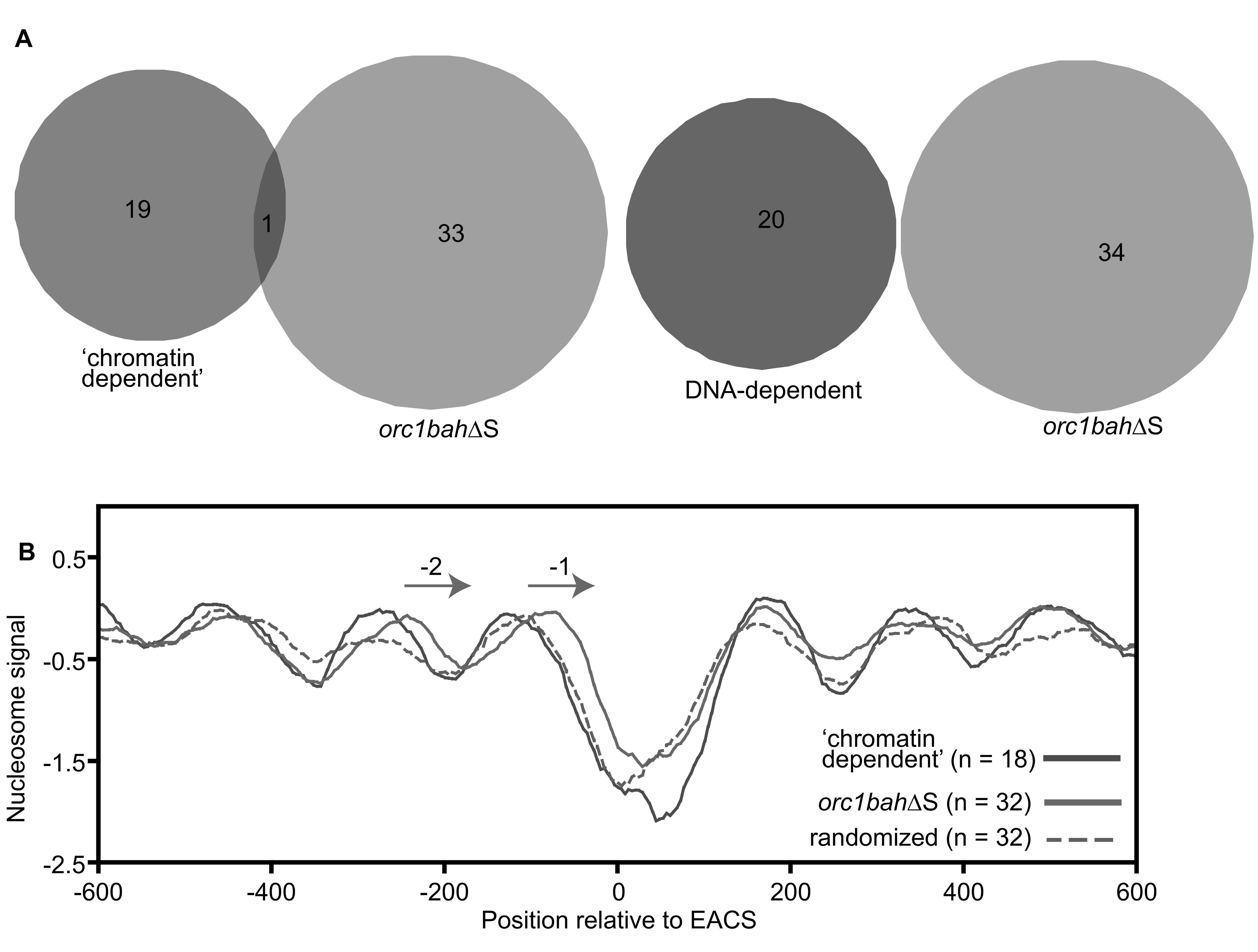

Supplement: Figure S5 — Origins requiring the Orc1BAH domain for ORC binding were distinct from ‘chromatin-dependent’ origins. In a previous study we defined a group of 34 origins that required the Orc1BAH domain for efficient binding by ORC in vivo (orc1bahΔ/ORC1 ratio</ = 0.3) [43]. (A) Venn diagrams indicating degree of overlap between the indicated origin groups. (B) Nucleosome configuration around the indicated groups of origins relative to the ORC binding site (EACS), as in Figure 5. (TIF) [file pgen.1003798.s005.tif]

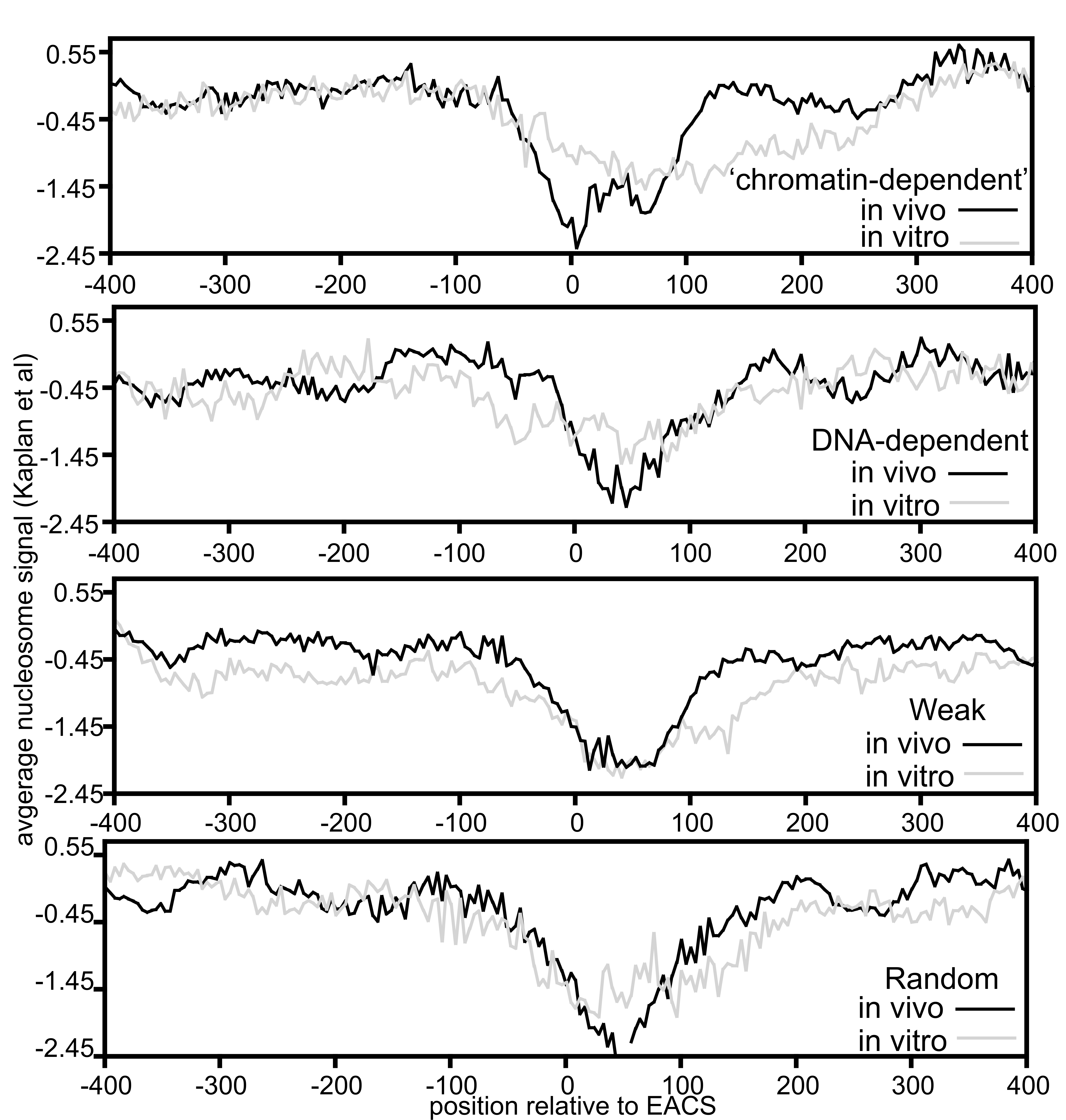

Supplement: Figure S6 — Comparison of nucleosome positioning in vitro and in vivo around the indicated origin groups; these analyses used nucleosome data from [62]. (TIF) [file pgen.1003798.s006.tif]

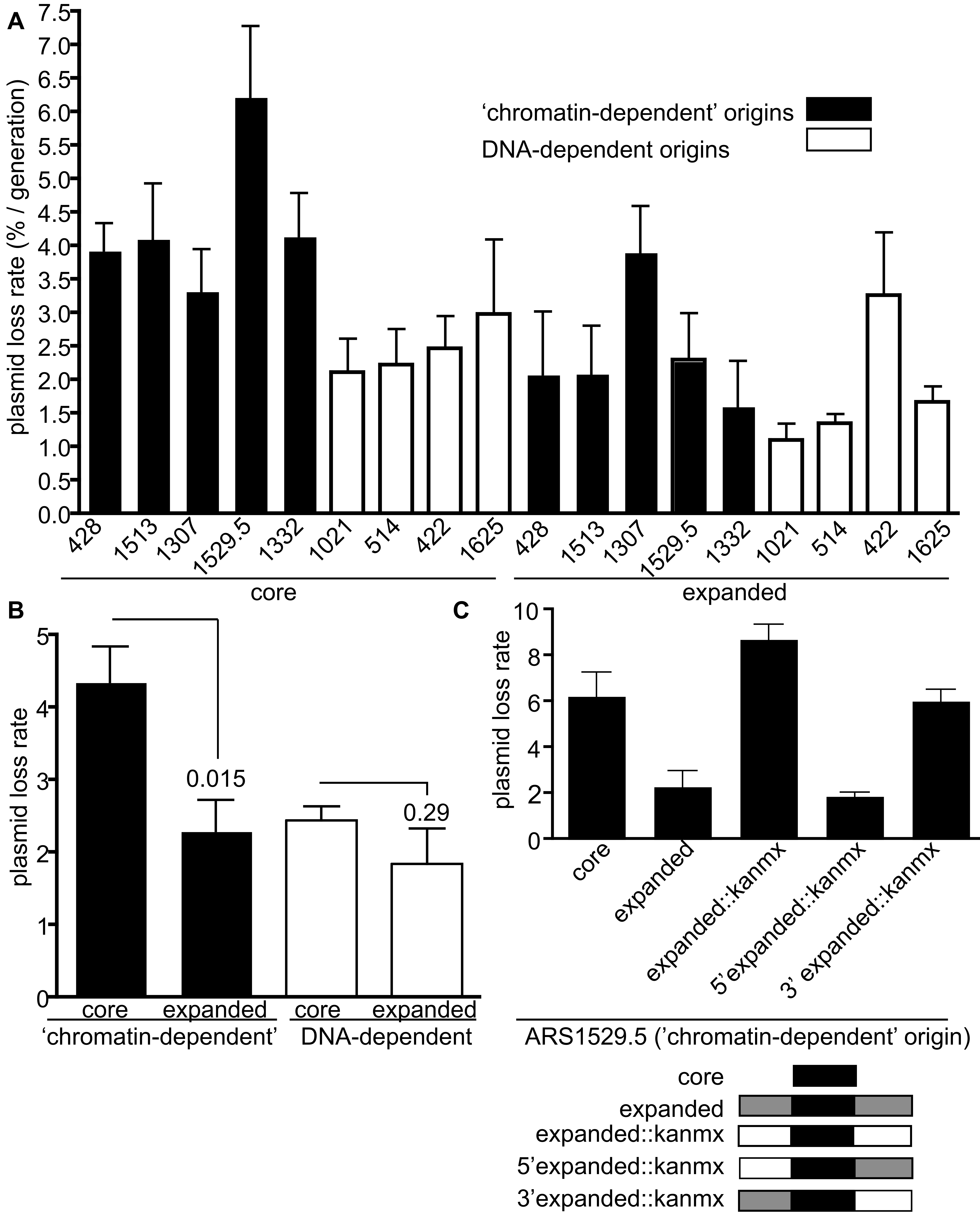

Supplement: Figure S7 — Functionality of ‘chromatin-dependent’ origins was enhanced by native sequences flanking the core origin, whereas DNA-dependent origins were comparably unaffected by such sequences. ‘Chromatin-dependent’ and DNA-dependent origins were distinguished based on the mechanisms that determined their ORC-origin interactions in vivo. While both groups of origins showed similar though not identical matches to the consensus ORC binding site (Figure S8), indicating that the basic ORC binding motif was maintained, they showed different local chromatin configurations that might be functionally relevant (Figure 5). To address whether these different binding mechanisms were associated with functional differences between these two classes of origins, we examined the ability of several origins from each group to replicate a plasmid. In particular, we reasoned that if ‘chromatin-dependent’ origins depended on a distinct local chromatin environment then they might be more sensitive to the presence of additional chromosomal sequences beyond the defined ‘core’ ARS compared to DNA-dependent origins. (A) To test this idea, the origin activity of several ‘chromatin-dependent’ and DNA dependent origins was compared using ARS assays. In one set of experiments, we assessed the activity of ‘core’ origins as defined by oriDB (Table S3). Given the arbitrary fragments used to define most ARSs, ‘core’ origins vary substantially in length. Therefore selection of origins for these analyses was in part based on their annotated lengths such that the mean core size for the ‘chromatin-dependent’ and DNA-dependent groups of origins did not differ substantially (‘chromatin-dependent’ core ARSs were 279+/−33 bp while DNA-dependent core ARSs were 293+/−43 bp). In the second set of experiments, the ARS activity of the ‘expanded origins’ was assessed. An expanded origin contained the core origin plus an additional 500 bp of flanking chromosomal sequence on either side of the ORC binding site. (B) On average, [file pgen.1003798.s007.tif]

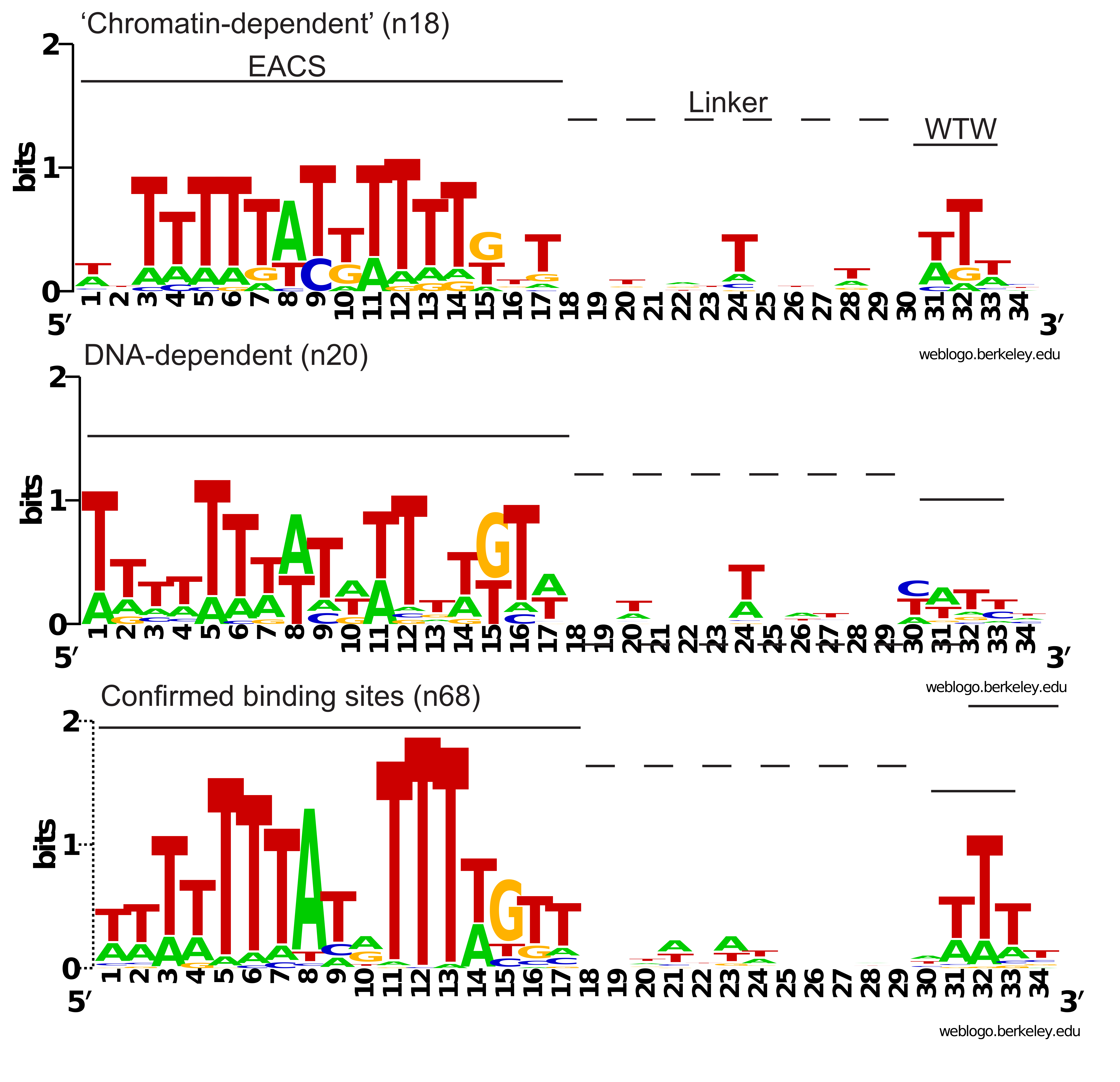

Supplement: Figure S8 — The consensus ORC binding site motifs derived from analyses of different origin groups. A consensus ORC binding site, including the 17 bp EACS, the WTW motif and the linker region in between these two elements was determined for ‘chromatin-dependent’ and DNA-dependent origins using Weblogo. These were compared to the consensus derived from 68 confirmed ORC binding sites (annotated and referenced on OriDB). The ORC binding sites were identified for 18 ‘chromatin-dependent’ origins and 20 DNA-dependent origins based on one of the following: a confirmed ACS, a match to an ORCACS, or a match to a proACS [38], [44], as in Figure 5. When necessary, annotated sequences were converted to a 34 bp binding site by including nucleotides 5′ and 3′ of the annotated ACS match. Sequences of ORC binding sites were aligned based on the start of the EACS on the T rich strand. (TIF) [file pgen.1003798.s008.tif]

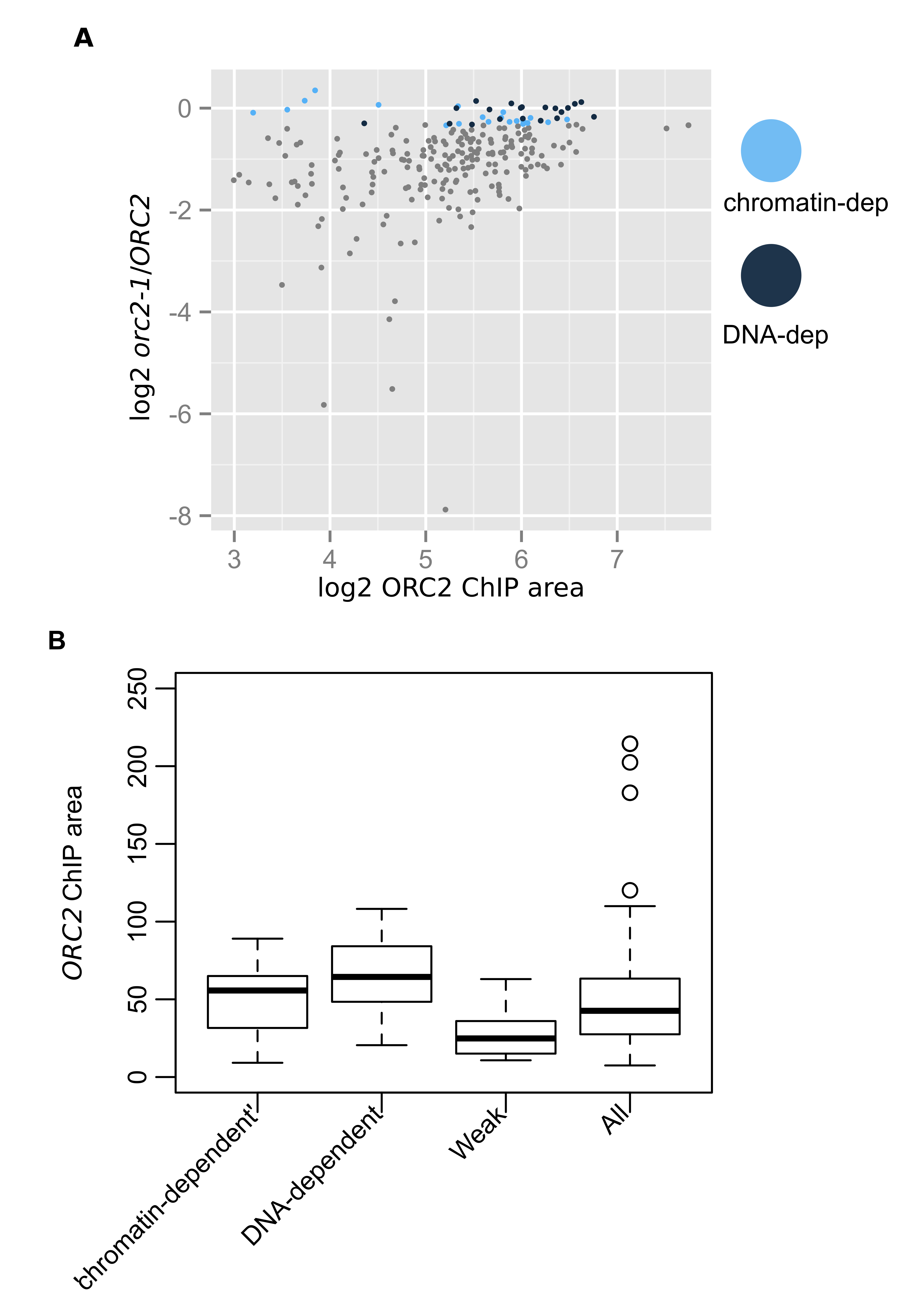

Supplement: Figure S10 — Chromatin-dependent origins are comprised of origins associated with virtually the full range of ORC peak sizes in the ORC2-ChIP-chip. A ‘chromatin-dependent’ origin was defined based on having a high orc2-1/ORC2 ratio binding peak ratio in ChIP (>/ = 0.8; orc2-1-resistant) but weak in vitro binding in the gEMSA (or, in some cases also a high Kd determined in EMSAs). A concern was that many such origins might have small ORC2 peaks and thus orc2-1-resistance might result from noise in the data. These control analyses suggest that was not a major concern. (A) A plot of the log2(orc2-1/ORC2) (y-axis) versus log2 of ORC2 peak area (x-axis) shows that the majority of ‘chromatin-dependent’ (blue) and DNA-dependent origins (black) corresponded to origins that generated similarly-sized ORC2 ChIP peak areas. Orc2-1-resistant origins and orc2-1-sensitive origins corresponded to origins that generated ORC2 ChIP peak areas across the entire range of peak areas measured. (B) Box-and-Whiskers plot of ORC2 ChIP peak areas for the indicated origin groups. The Box-and-Whiskers plots are as described in Figure 5. (TIF) [file pgen.1003798.s010.tif]

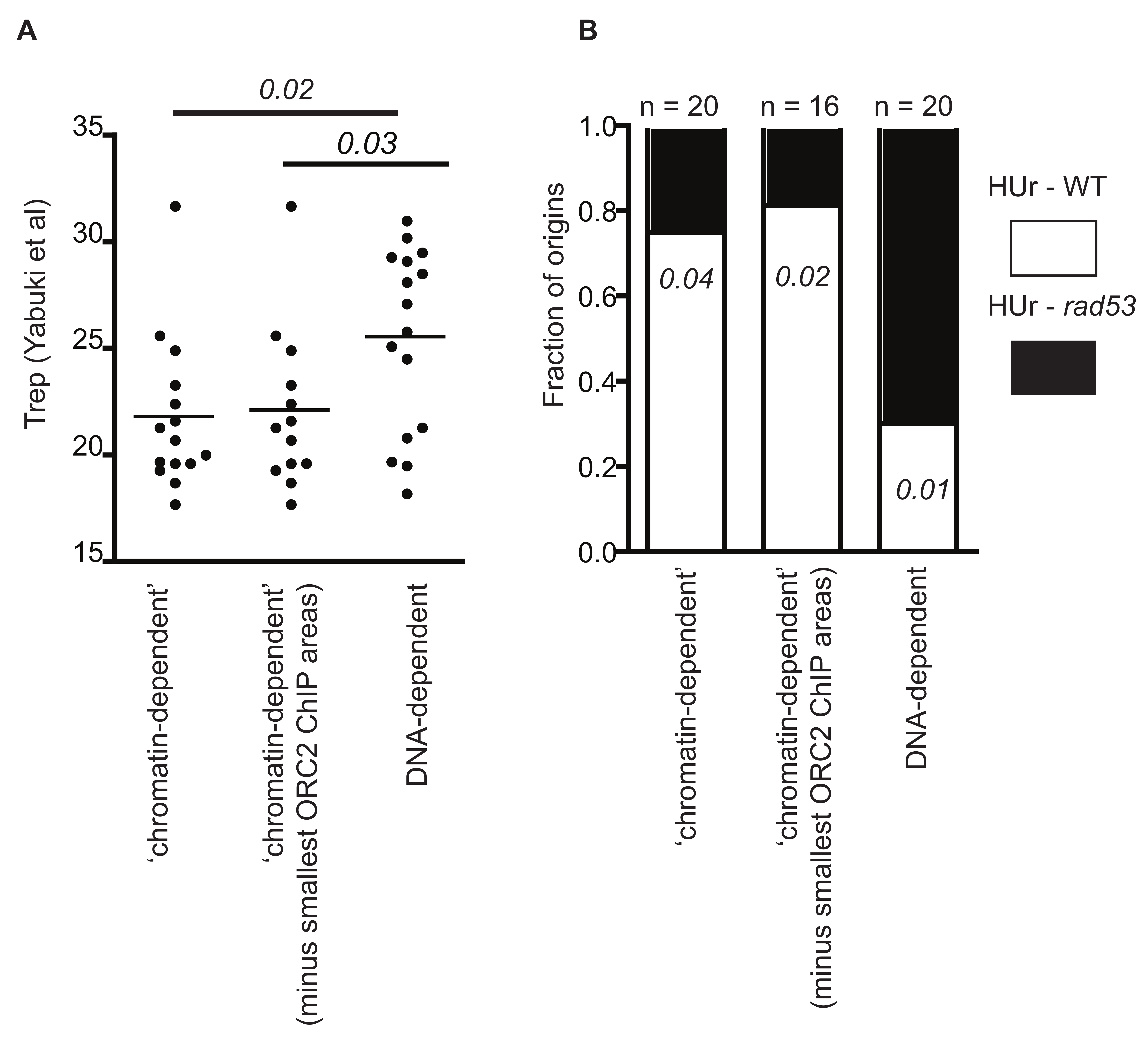

Supplement: Figure S11 — Timing and HU-resistance differences between ‘chromatin-dependent’ and DNA-dependent origins determined after removal of the four ‘chromatin-dependent’ origins associated with the smallest ORC2 peak areas. (A) This plot is analogous to the one shown in Figure 6A of the main text. Two of ‘chromatin-dependent’ origins removed prior to these analyses had early Treps, but two were not assigned Trep values. Hence removal of all four of these origins had a negligible effect on the average Trep differences between ‘chromatin-dependent’ and DNA-dependent origins. (B) This plot is analogous to the one shown in Figure 6B of the main text. There was no substantial change in the difference between ‘chromatin-dependent’ and DNA-dependent origins in terms of HU-resistant activation after removal of the four smallest ‘chromatin-dependent’ origins. P-values indicating the significance of the differences in distribution of origin types in each group relative to all origins indicated. (TIF) [file pgen.1003798.s011.tif]

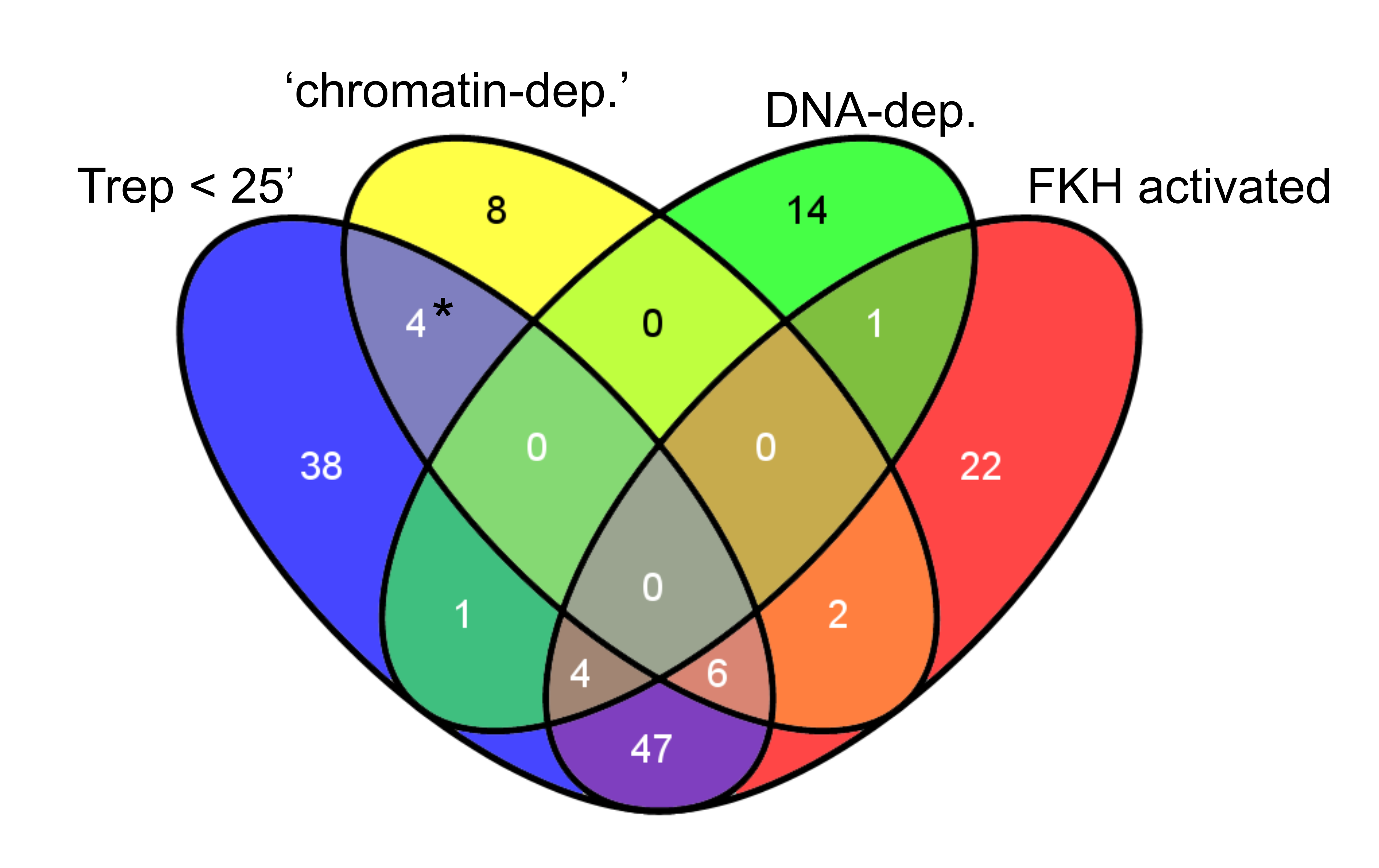

Supplement: Figure S12 — Analyses of the relationship between FKH regulation and origin-binding mechanisms by ORC. Overlap among key groups (Venn diagrams) of origins is shown for: Early origins Trep (Trep<25′) that were also identified in our ORC ChIP study (n = 100) [68]; Fkh1/2 activated origins (n = 82) (57 origins with Trep<25′ were Fkh1/2 activated) [22]; ‘chromatin-dependent’ origins (n = 20); and DNA-dependent origins (n = 20). Of the 10 ‘chromatin-dependent’ origins that are fire early in S-phase, six of these are Fkh-activated, while four were not regulated by Fkh1/2 were not regulated. 43 origins out of the 100 that fire early (Trep<25′) were not called as Fkh-activated. (TIF) [file pgen.1003798.s012.tif]
